# Lead-I ECG for detecting atrial fibrillation in patients attending primary care with an irregular pulse using single-time point testing: A systematic review and economic evaluation

**DOI:** 10.1371/journal.pone.0226671

**Published:** 2019-12-23

**Authors:** Rui Duarte, Angela Stainthorpe, James Mahon, Janette Greenhalgh, Marty Richardson, Sarah Nevitt, Eleanor Kotas, Angela Boland, Howard Thom, Tom Marshall, Mark Hall, Yemisi Takwoingi

**Affiliations:** 1 Liverpool Reviews and Implementation Group, University of Liverpool, Liverpool, United Kingdom; 2 Health Economics and Outcomes Research Ltd, Cardiff, United Kingdom; 3 Coldingham Analytical Services, Berwickshire, United Kingdom; 4 York Health Economics Consortium, University of York, York, United Kingdom; 5 Bristol Medical School: Population Health Sciences, University of Bristol, Bristol, United Kingdom; 6 Institute of Applied Health Research, University of Birmingham, Birmingham, United Kingdom; 7 Liverpool Heart and Chest Hospital, Liverpool, United Kingdom; 8 NIHR Birmingham Biomedical Research Centre, University Hospitals Birmingham NHS Foundation Trust and University of Birmingham, Birmingham, United Kingdom; University of Oxford, UNITED KINGDOM

## Abstract

**Background:**

Atrial fibrillation (AF) is the most common type of cardiac arrhythmia and is associated with increased risk of stroke and congestive heart failure. Lead-I electrocardiogram (ECG) devices are handheld instruments that can detect AF at a single-time point.

**Purpose:**

To assess the diagnostic test accuracy, clinical impact and cost effectiveness of single-time point lead-I ECG devices compared with manual pulse palpation (MPP) followed by a 12-lead ECG for the detection of AF in symptomatic primary care patients with an irregular pulse.

**Methods:**

Electronic databases (MEDLINE, MEDLINE Epub Ahead of Print and MEDLINE In-Process, EMBASE, PubMed and Cochrane Databases of Systematic Reviews, Cochrane Central Database of Controlled Trials, Database of Abstracts of Reviews of Effects, Health Technology Assessment Database) were searched to March 2018. Two reviewers screened the search results, extracted data and assessed study quality. Summary estimates of diagnostic accuracy were calculated using bivariate models. Cost-effectiveness was evaluated using an economic model consisting of a decision tree and two cohort Markov models.

**Results:**

**Diagnostic accuracy**

The diagnostic accuracy (13 publications reporting on nine studies) and clinical impact (24 publications reporting on 19 studies) results are derived from an asymptomatic population (used as a proxy for people with signs or symptoms of AF). The summary sensitivity of lead-I ECG devices was 93.9% (95% confidence interval [CI]: 86.2% to 97.4%) and summary specificity was 96.5% (95% CI: 90.4% to 98.8%).

**Cost effectiveness**

The de novo economic model yielded incremental cost effectiveness ratios (ICERs) per quality adjusted life year (QALY) gained. The results of the pairwise analysis show that all lead-I ECG devices generate ICERs per QALY gained below the £20,000-£30,000 threshold. Kardia Mobile is the most cost effective option in a full incremental analysis. Lead-I ECG tests may identify more AF cases than the standard diagnostic pathway. This comes at a higher cost but with greater patient benefit in terms of mortality and quality of life.

**Limitations:**

No published data evaluating the diagnostic accuracy, clinical impact or cost effectiveness of lead-I ECG devices for the target population are available.

**Conclusions:**

The use of single-time point lead-I ECG devices in primary care for the detection of AF in people with signs or symptoms of AF and an irregular pulse appears to be a cost effective use of NHS resources compared with MPP followed by a 12-lead ECG, given the assumptions used in the base case model.

**Registration:**

The protocol for this review is registered on PROSPERO as CRD42018090375.

## Introduction

Atrial fibrillation (AF) is a disturbance in heart rhythm (arrhythmia) caused by abnormal electrical activity in the upper chambers of the heart (atria).[1] AF is the most common type of arrhythmia. Estimates from 2010 suggest that 20.9 million men and 12.6 million women worldwide are living with AF.[2] The median age of diagnosis is 75 years with the highest number of cases between the ages of 75 to 79 years in males and 80 to 84 years in females.[3]

AF can be paroxysmal (intermittent episodes lasting less than 7 days that stop without treatment), persistent (episodes lasting longer than 7 days and do not terminate without treatment) or permanent (present all the time). AF can be categorised as valvular or non-valvular depending on the underlying cause (i.e. whether valve disease is present or not). Both valvular and non-valvular AF can be paroxysmal, persistent or permanent.[4] Patients diagnosed with paroxysmal AF may develop persistent or permanent AF.[2] It is possible, but unusual, for some people with persistent AF to revert to normal sinus rhythm.[2]

Patients with AF may experience palpitations, dizziness, shortness of breath and tiredness. However, AF can be asymptomatic and may only be identified when people attend medical appointments for other conditions. Due to its intermittent nature, many cases of paroxysmal AF remain undiagnosed.[2] Cases of paroxysmal AF may only be detected with prolonged monitoring, rather than by a single examination.[2]

The National Institute for Health and Care Excellence (NICE)[5] recommends that, after positive manual pulse palpation (MPP), an AF diagnosis should be confirmed with an electrocardiogram (ECG). People who present to primary care with signs or symptoms of AF and an irregular pulse should be referred for a 12-lead ECG in the days following their primary care appointment if a 12-lead ECG is not available in the practice. Treatment (where indicated) begins following the results of the 12-lead ECG test. Lead-I ECG devices are handheld instruments that can be used to detect AF. Lead-I ECGs are so-called because of the 12-lead ECG that they simulate (i.e. Lead-I) rather than the fact that they record "one lead" only. They could be used to detect AF during a primary care appointment in people who present with signs or symptoms and have an irregular pulse, which may reduce the time to initiating anticoagulation therapy.

## Objectives

The aim of this study was to assess the diagnostic test accuracy, the clinical impact and the cost effectiveness of single-time point lead-I ECG devices for the detection of AF in people presenting to primary care with signs or symptoms of AF and who have an irregular pulse, compared with MPP followed by a 12-lead ECG in primary or secondary care (prior to initiation of anticoagulation therapy). To achieve this aim we:

1. conducted systematic reviews of the diagnostic accuracy and clinical impact of lead-I ECG devices for (1) detecting AF in people presenting to primary care with signs or symptoms of AF, or, if evidence was not available for this population/setting, for (2) detecting AF in an asymptomatic population defined as people presenting to any setting without symptoms of AF, with or without a previous diagnosis of AF
2. developed an economic model to assess the cost effectiveness of single-time point lead-I ECG devices compared with MPP followed by a 12-lead ECG in primary or secondary care in people presenting to primary care with signs or symptoms of AF who have an irregular pulse.

## Methods: Assessment of clinical impact and diagnostic test accuracy

The systematic review methods followed the general principles outlined in the Centre for Reviews and Dissemination (CRD) guidance for conducting reviews in health care,[6] the NICE Diagnostics Assessment Programme manual[7] and the Cochrane Handbook for Systematic Reviews of Diagnostic Test Accuracy.[8] The systematic review was conducted according to a prespecified protocol[4] and is registered on PROSPERO as CRD42018090375. The systematic review is reported in accordance with the Preferred Reporting Items for Systematic Reviews and Meta-Analyses (PRISMA) for diagnostic test accuracy (DTA) studies.[9] The PRISMA-DTA checklist and PRISMA-DTA for abstracts checklist are presented in S1 and S2 Tables respectively.

### Data sources and searches

Electronic databases (MEDLINE, MEDLINE Epub Ahead of Print and MEDLINE In-Process, EMBASE, PubMed and Cochrane Databases of Systematic Reviews, Cochrane Central Database of Controlled Trials, Database of Abstracts of Reviews of Effects, Health Technology Assessment Database) were searched up to 9^th^ March 2018. The search strategy used for the MEDLINE database is presented in S1 Text. The MEDLINE search strategy was adapted to enable similar searches of the other relevant electronic databases.

The search results were managed using EndNote X8 software. The reference lists of relevant systematic reviews and eligible studies were hand-searched to identify further potentially relevant studies.

### Study selection

The citations identified were assessed for inclusion in the review using a two-stage process. First, two reviewers independently screened all titles and abstracts identified by the electronic searches to identify potentially relevant articles to be retrieved. Second, full-text copies of these studies were obtained and assessed independently by two reviewers for inclusion using the eligibility criteria outlined in S3 Table. Any disagreements were resolved through discussion at each stage, and, if necessary, in consultation with a third reviewer. Studies that assessed the diagnostic accuracy of lead-I ECG devices used at a single-time point to detect AF in an asymptomatic population were considered for inclusion due to the absence of studies in symptomatic populations. We considered an asymptomatic population to comprise people not presenting with symptoms of AF, with or without a previous diagnosis of AF.

### Data extraction

Data were extracted relating to the information described in S3 Table. Data extraction was carried out by one reviewer and checked for accuracy by a second reviewer. Any disagreements were resolved through discussion, and, if necessary, in consultation with a third reviewer.

### Quality assessment

The methodological quality of the included diagnostic accuracy studies was assessed using the QUality Assessment of Diagnostic Accuracy Studies—2 (QUADAS-2) tool tailored to the review question.[10] The methodological quality of cross-sectional and case-controlled studies evaluating the clinical impact of lead-I ECG devices was assessed using the Newcastle-Ottawa quality assessment scale.[11, 12]

Quality assessment of the included studies was undertaken by one reviewer and checked by a second reviewer. Any disagreements were resolved by discussion, and, if necessary, in consultation with a third reviewer.

### Data synthesis and analysis

The sensitivity and specificity of each index test were summarised in forest plots and plotted in receiver operating characteristic (ROC) space. Pooled estimates of sensitivity and specificity with 95% confidence intervals (CIs) were obtained using bivariate models.[13] The bivariate model was fitted using the metandi and xtmelogit commands in Stata version 14. Summary receiver operating characteristic (SROC) plots were produced using RevMan 5.3. When there were few studies, the bivariate model was reduced to two univariate random effect logistic regression models by assuming no correlation between sensitivity and specificity across studies.[14] When little or no heterogeneity was observed on forest plots and SROC plots, the models were further simplified into fixed effect models by eliminating the random effects parameters for sensitivity and/or specificity.[14] Judgement of heterogeneity was based on the visual appearance of forest plots and SROC plots in addition to clinical judgement regarding potential sources of heterogeneity.

The analyses were stratified by whether diagnosis of AF was made by a trained healthcare professional interpreting the lead-I ECG trace, or by the lead-I ECG algorithm. For both sets of analyses, the reference standard was interpretation of the 12-lead ECG trace by a trained healthcare professional. When studies reported data for two types of lead-I ECG device and two different interpreters, one dataset was chosen and sensitivity analyses were performed using the alternative datasets. Clinical impact outcomes were synthesised narratively.

## Methods: Assessment of cost effectiveness

A de novo economic analysis was undertaken following the diagnostic pathway for patients presenting to primary care with signs or symptoms of AF and an irregular pulse. Results were presented over a time horizon of 30 years with patients entering the model at age 70. The economic evaluation took a NHS/Personal Social Services (PSS) perspective. The economic evaluation is only relevant to primary care practices where patients have to wait at least 48 hours between an initial consultation with the GP and having a 12-lead ECG; this allows the benefit of early anticoagulation and rate control treatment for those patients who receive a positive lead-I ECG to be considered. The base case model assumptions are presented in S5 Table.

### Model structure

A decision tree and two cohort Markov models were built in Microsoft Excel® (Microsoft Corporation, Redmond, WA, USA). The decision tree describes the pathway that a patient presenting to primary care with signs or symptoms of AF and an irregular pulse follows in the initial GP consultation (S1–S3 Figs). The first Markov model captured the differences in the costs and benefits of treatment (standard diagnostic pathway outlined in NICE CG180 versus lead-I ECG pathway) during the first 3 months after the initial appointment (S4 Fig). During this period, some patients will be diagnosed with AF and start treatment whilst other patients will have further tests to diagnose or rule out AF (where ‘rule out’ means no diagnosis of AF is recorded and no treatment for AF is started). The second Markov model captured the differences in lifetime costs and benefits after patients have either received a diagnosis of AF or have had AF ruled out (S5 Fig). Patients remained in the second Markov model until death. The cycle length was 3 months in the second Markov model. Costs and benefits were discounted at 3.5% per year.

### Patient population

The modelled population was people with signs or symptoms of AF plus an irregular pulse. This population includes patients with AF and patients without AF who are similarly symptomatic. Estimates of the prevalence of AF by age and sex were taken from a paper by Adderley;[15] these age-sex specific prevalence estimates are based on the results of a study carried out using primary care records from UK general practice in 2016.

The proportion of patients with AF who are symptomatic was taken from an observational cohort study of data from the US Outcomes Registry for Better Informed Treatment of Atrial Fibrillation (ORBIT-AF registry) by Piccini;[16] the study reports that women with AF were more likely to be symptomatic than men (67.9% versus 57.5%).

The proportion of patients with symptomatic undiagnosed AF who have paroxysmal AF could not be found in the literature. A fixed-effects meta-analysis published by Welton[17] reported that the proportion of patients with paroxysmal AF (not explicitly symptomatic) varied substantially between the studies[18–20] included in the meta-analysis (from 0.059 to 0.835). Given the wide range reported by Welton[17] and the lack of evidence specifically on incidence rates for symptomatic paroxysmal AF, it was assumed in our base case that 50% of patients in the model with AF would have paroxysmal AF.

### Tests and treatments

Cost per lead-I ECG test was calculated as the annual cost per device divided by the number of patients in the eligible population per year plus any extra costs associated with each use of the device (S5A and S5B Table). Costs for the 12-lead ECG tests were estimated using a microcosting approach for 12-lead ECG tests carried out in primary care and using NHS Reference Costs for tests carried out in secondary care (S6 Table). The proportion of patients receiving anticoagulation for AF was estimated using data from the Quality and Outcomes Framework 2016/2017 (AF007).[21] Apixaban was used as the basis for modelling costs and outcomes for all patients receiving anticoagulant therapy.

### Mortality, cardiovascular events and adverse events

Age- and sex-adjusted mortality rates were estimated for patients with and without AF who were and were not receiving anticoagulant therapy, and who had not experienced a previous cardiovascular event (CVE) (S7 Table). Mortality risk for patients who experienced a subsequent CVE was assumed to be 2.6 times greater than mortality risk for patients with no history of CVEs, based on the results of a study of stroke survivors in Norway.[22] The CVEs included in the model were: ischaemic stroke, transient ischaemic attack and haemorrhagic stroke. Clinically relevant bleeds were considered to be adverse events (AEs). Rates for CVEs depended on AF- and treatment status, and whether a patient had experienced a previous CVE. Rates for AEs depended on AF- and treatment status but did not take account of the history of previous events (S8A–S8E Table).

### Utilities

Utility values for the symptomatic and asymptomatic AF-positive population were based on a study by Berg.[23] Berg provides the coefficients of regression models fitted to the results of the EQ-5D-3L[24] questionnaire completed as part of a large European survey of patients with AF. Mean age-specific utility values for symptomatic patients with AF were calculated using the coefficients from the study by Berg[23] and adjusted for model age, sex ratio and symptom proportions.

### Analysis of uncertainty

Probabilistic sensitivity analysis (PSA) results were presented to reflect uncertainty in the model inputs; extensive deterministic sensitivity analysis and scenario analysis were also carried out to assess the impact of uncertainty in model assumptions. We report the total costs of the annual number of symptomatic patients with positive MPP seen by a single GP, total quality adjusted life years (QALYs) for these patients, incremental costs and QALYs, and incremental cost effectiveness ratios (ICERs).

## Results

**Fig 1:**
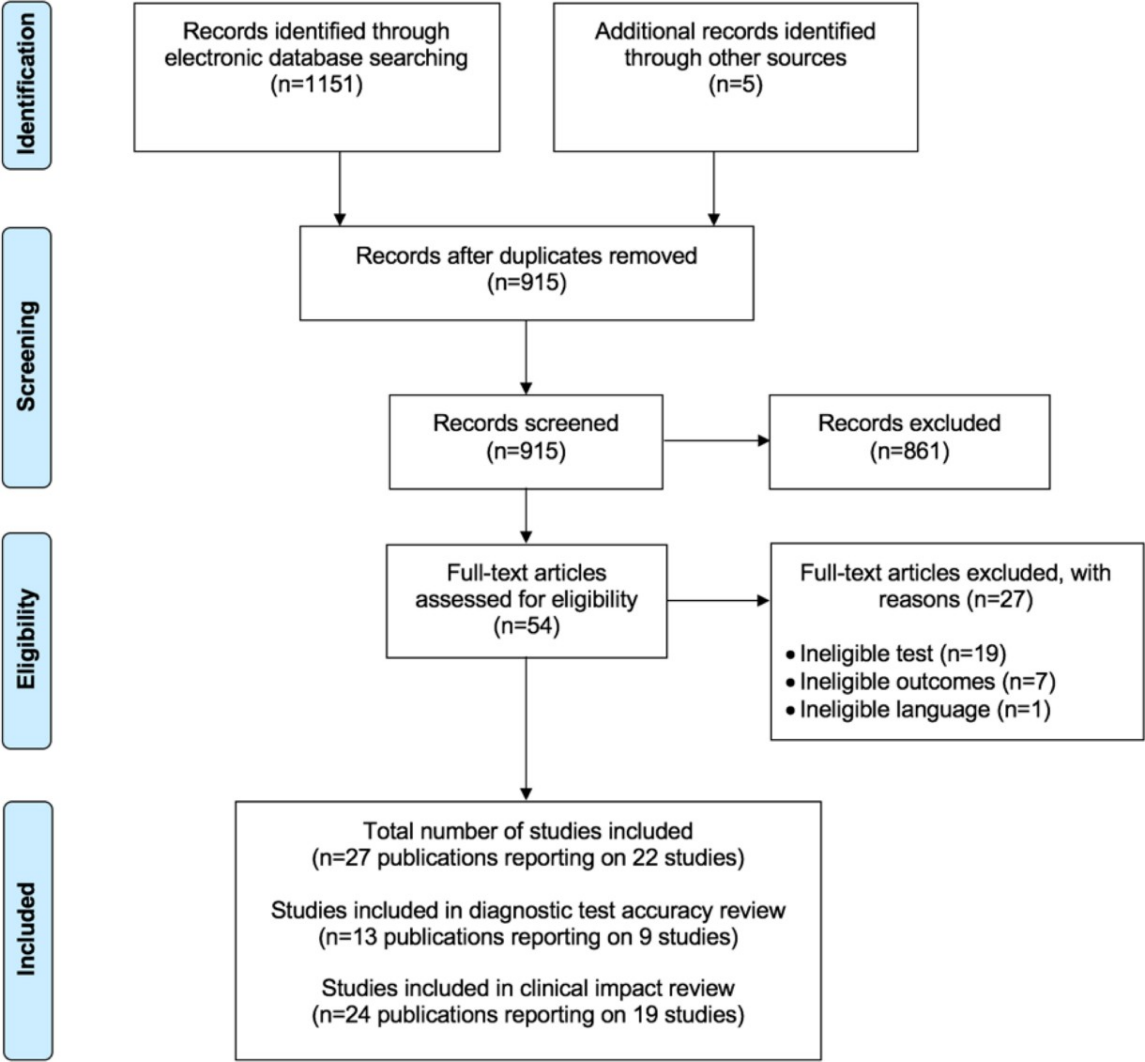
PRISMA flow chart.

The electronic database searches identified 1151 citations (915 unique records). No studies were identified for the population of interest (i.e. people with signs or symptoms of AF and who have an irregular pulse). Therefore, all the studies included in the systematic reviews assessed the diagnostic accuracy and clinical impact of lead-I ECG devices used at a single-time point to detect AF were performed in an asymptomatic population. The PRISMA[25] flow chart detailing the screening process for the review is shown in Fig 1. Studies excluded at the full-text paper screening stage with reasons for exclusion are presented in S2 Text.

### Diagnostic test accuracy

We identified 13 publications[26–38] reporting on nine studies. In these studies, the index test (lead-I ECG device) was interpreted by the device algorithm or by a trained healthcare professional, including cardiologists, electrophysiologists and general practitioners. All studies used a 12-lead ECG device interpreted by a trained healthcare professional as the reference standard. The characteristics of the nine included diagnostic test accuracy studies are summarised in Table 1. All studies were assessed for risk of bias and applicability using the QUADAS-2 tool[10] and a summary of the results is presented in S9 Table.

**Table 1:** Characteristics of studies included in the diagnostic test accuracy review.

Study	Study design; country and setting	Population; number in analysis and recruitment details	Age; sex and risk factors for AF	Lead-I ECG device	Interpreter of lead-I ECG	Test sequence
Crockford 2013[37]	Cross-sectional; UK; secondary care	Patients referred to an electrophysiology department; N = 176; NR	Age; sex and risk factors: NR	RhythmPad GP	Algorithm	12-lead ECG followed by lead-I ECG
Desteghe 2017[28]	Case-control; Belgium; tertiary care	Inpatients at cardiology ward; N = 265; NR	Mean age ± SD (years): 67.9 ± 14.6 Sex: 138 (43.1%) female Pacemaker: 4/55 (7.3%) were intermittently paced, and 18/55 (32.7%) were not being paced during the recordings Known AF: 114/320 (35.6%) AF at time of study: 11.9% on 12-lead ECG; 3.4% of all patients admitted because of symptomatic AF Paroxysmal AF: 54.4%	MyDiagnostick and Kardia Mobile	Algorithm and two electrophysiologists (results presented separately for algorithm and two electrophysiologists)	12-lead ECG followed by lead-I ECG (order for the use of the different lead-I ECG tests not specified)
Doliwa 2009[29]	Case-control; Sweden; secondary care	People with AF, atrial flutter or sinus rhythm; N = 100; patients were recruited from a cardiology outpatient clinic	Age; sex and risk factors: NR	Zenicor-ECG	Cardiologist	12-lead ECG followed by lead-I ECG
Haberman 2015[31]	Case-control; USA; community and secondary care	Healthy young adults, elite athletes and cardiology clinic patients; N = 130; NR*	Mean age ± SD (years): 59 ± 15 Sex: 73 (56%) male Risk factors: NR	Kardia Mobile	Electrophysiologist	Lead-I ECG followed by 12-lead ECG
Koltowski 2017[38]	Cross-sectional; Poland; tertiary care	Patients in a tertiary care centre; N = 100; NR	Age; sex and risk factors: NR	Kardia Mobile	Cardiologist	Lead-I ECG followed by 12-lead ECG
Lau 2013[33]	Case-control; Australia; secondary care	Patients at cardiology department; N = 204; NR	Age and sex: NR Known AF: 48 (24%)	Kardia Mobile	Algorithm	Lead-I ECG followed by 12-lead ECG
Tieleman 2014[34]	Case-control; Netherlands; secondary care	Patients with known AF and patients without a history of AF attending an outpatient cardiology clinic or a specialised AF outpatient clinic; N = 192; random selection of patients due to have a 12-lead ECG	Mean age ± SD (years): 69.4 ± 12.6 Sex: 48.4% male Risk factors: NR	MyDiagnostick	Algorithm	Lead-I ECG followed by 12-lead ECG
Vaes 2014[35]	Case-control; Belgium; primary care	Patients with known AF and patients without a history of AF; N = 181; GP invitation	Mean age ± SD (years): 74.6 ± 9.7 Sex: 91 (48%) female Known AF: 151 (83.4%)	MyDiagnostick	Algorithm	Lead-I ECG followed by 12-lead ECG
Williams 2015[36]	Case-control; UK; secondary care	Patients with known AF attending an AF clinic and patients with AF status unknown who were attending the clinic for non-AF related reasons; N = 95; patients attending clinic appointments who were due to have a 12-lead ECG	Age; sex and risk factors: NR	Kardia Mobile	Cardiologist and general practitioner with an interest in cardiology	12-lead and lead-I ECG carried out simultaneously

AF = atrial fibrillation; ECG = electrocardiogram; GP = general practice; NR = not reported; SD = standard deviation

*Community population not included in the analysis as these comprised healthy young adults and elite athletes; only secondary care patients were included in the analysis

### Interpreter of lead-I ECG: Trained healthcare professional

**Fig 2:**
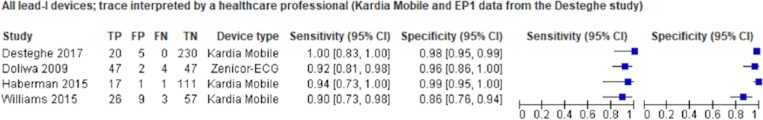
Forest plot of individual studies included in the meta-analysis of all lead-I ECG devices (trace interpreted by a trained healthcare professional). CI = confidence interval; EP1 = electrophysiologist 1; FN = false negative; FP = false positive; TN = true negative; TP = true positive.

Data from four studies[28, 29, 31, 36] contributed to the meta-analyses (two studies of Kardia Mobile,[31, 36] one study of Zenicor-ECG[29] and one study of MyDiagnostick and Kardia Mobile).[28] The main meta-analysis (number of AF cases = 118, total N = 580), indicated that the pooled sensitivity of lead-I ECG devices was 93.9% (95% CI: 86.2% to 97.4%) and pooled specificity was 96.5% (95% CI: 90.4% to 98.8%) (Fig 2). The SROC plot which shows the individual study results as well as the meta-analysis result is presented in S6A Fig. Across the sensitivity analyses, numerical results were similar; pooled sensitivity values ranged from 89.8% to 94.3% and pooled specificity values ranged from 95.6% to 97.4%.

### Interpreter of lead-I ECG: Algorithm

Data from four studies[28, 33–35] were included in the meta-analyses (two studies of MyDiagnostick,[34, 35] one study of Kardia Mobile,[33] and one study MyDiagnostick and Kardia Mobile).[28] Meta-analysis (number of AF cases = 219, total N = 842) showed a pooled sensitivity of 96.2% (95% CI: 86.0% to 99.0%) and pooled specificity was 95.2% (95% CI: 92.9% to 96.8%). SROC plot is presented in S6B Fig. Numerical results were similar across the sensitivity analyses; pooled sensitivity values ranged from 88.0% to 96.2% and pooled specificity values ranged from 94.4% to 97.2%.

A summary of the results from the meta-analyses are presented in Table 2.

**Table 2:** Results from meta-analyses of lead-I ECG devices.

Data input from the Desteghe* and Williams** studies	Lead-I ECG device (# studies) in the meta-analyses	# AF cases	N	Pooled sensitivity (95% CI)	Pooled specificity (95% CI)
**Lead-I ECG trace interpreted by a trained healthcare professional (main analysis)**					
Kardia Mobile device and EP1* and cardiologist** data	Kardia Mobile (3), Zenicor-ECG (1)	118	580	93.9% (86.2% to 97.4%)	96.5% (90.4% to 98.8%)
Lead-I ECG trace interpreted by a trained healthcare professional (sensitivity analyses, cardiologist data**)					
MyDiagnostick device and EP1* data	Kardia Mobile (2), Zenicor-ECG (1), MyDiagnostick (1)	118	582	90.8% (83.8% to 95.0%)	95.6% (89.4% to 98.3%)
MyDiagnostick device and EP2 data	Kardia Mobile (2), Zenicor-ECG (1), MyDiagnostick (1)	118	582	89.8% (82.7% to 94.1%)	96.8% (90.6% to 99.0%)
Kardia Mobile device and EP2* data	Kardia Mobile (3), Zenicor-ECG (1)	120	584	91.8% (85.1% to 95.7%)	97.1% (90.8% to 99.1%)
Lead-I ECG trace interpreted by a trained healthcare professional (sensitivity analyses, GP data**)					
Kardia Mobile device and EP1* and GP** data	Kardia Mobile (3), Zenicor-ECG (1)	118	580	94.3% (87.9% to 97.4%)	96.0% (85.4% to 99.0%)
**Lead-I ECG trace interpreted by a trained healthcare professional (sensitivity analyses, Kardia Mobile)**					
Kardia Mobile device and EP1* data	Kardia Mobile (3)	67	480	94.0% (85.1% to 97.7%)	96.8% (88.0% to 99.2%)
Kardia Mobile device and EP2* data	Kardia Mobile (3)	69	484	91.3% (82.0% to 96.0%)	97.4% (88.3% to 99.5%)
**Lead-I ECG trace interpreted by lead-I ECG device algorithm alone**					
MyDiagnostick device* data	Kardia Mobile (1), MyDiagnostick (3)	219	842	96.2% (86.0% to 99.0%)	95.2% (92.9% to 96.8%)
Kardia Mobile device* data	Kardia Mobile (2), MyDiagnostick (2)	219	842	95.3% (70.4% to 99.4%)	96.2% (94.2% to 97.6%)
MyDiagnostick device only	MyDiagnostick (3)	171	638	95.2% (79.0% to 99.1%)	94.4% (91.9% to 96.2%)
Kardia Mobile device only	Kardia Mobile (2)	70	469	88.0% (32.3% to 99.1%)	97.2% (95.1% to 98.5%)

# = number of; AF = atrial fibrillation; CI = confidence interval; EP1 = electrophysiologist 1; EP2 = electrophysiologist 2; GP = general practitioner

*From the Desteghe study^27^

**From the Williams study^35^

### Clinical impact

We identified 24 publications[26–34, 38–52] reporting on 19 studies with a total of 33,993 participants. The index tests evaluated included ImPulse (one study),[50] Kardia Mobile (12 studies), [31, 33, 38, 40, 41, 43, 44, 46, 48, 49, 51, 52] MyDiagnostick (four studies),[34, 39, 45, 47] Zenicor ECG (one study)[29] and MyDiagnostick and Kardia Mobile (one study).[28] Test failure rate was reported in nine studies[28, 31, 39, 43, 44, 47–50] and ranged from 0.1% to 9%. Results for test failure rate included both failure of the lead-I ECG algorithm to produce a result and poor quality of the lead-I ECG trace. Diagnostic yield was reported in 13 studies.[28, 34, 39, 41, 43–49, 51, 52] The percentage of new patients diagnosed with AF ranged from 0.4% to 5.8%. Data for this outcome were considered too heterogeneous for a pooled estimate to be clinically meaningful. Only one study[28] reported the concordance between lead-I ECG devices (Kardia Mobile and MyDiagnostick) observing no difference in agreement between the devices. Two studies[46, 48] reported a change in treatment management following the use of the Kardia Mobile lead-I ECG in new patients diagnosed with AF. Acceptability of lead-I ECG devices was reported in four studies,[41, 45, 46, 49] with generally positive views from patients and healthcare staff. Full clinical impact results and quality assessment of studies included is presented in the study monograph.[53]

### Cost effectiveness

Four base case scenarios were investigated to estimate cost effectiveness depending on the waiting times for a 12-lead ECG test (2 days or 14 days) and the location of the 12-lead ECG test (primary or secondary care). Pairwise cost effectiveness results assuming the 12-lead ECG was carried out in primary care and 2 days to 12-lead ECG (Base Case 1) for each index test versus the standard diagnostic pathway are presented in Table 3 and incremental analysis results are shown in Table 4. Costs and QALYs generated in Base Case 1 are shown in S10A and S10B Table. Results for the other three base case scenarios are presented in S11A–S11L Table.

**Table 3:** Base Case 1: Pairwise cost effectiveness analysis.

Strategy	Costs	QALYs	Incremental costs	Incremental QALYs	ICER/ QALY gained
Standard pathway	£514,187	447.963			
Kardia Mobile	£515,551	449.249	£1,364	1.286	£1,060
imPulse	£530,745	448.987	£16,557	1.024	£16,165
MyDiagnostick	£521,233	449.024	£7,046	1.061	£6,638
Generic lead-I device	£516,730	449.246	£2,543	1.284	£1,981
Zenicor-ECG	£518,468	449.199	£4,281	1.236	£3,462
RhythmPad GP*	£518,436	448.573	£4,249	0.610	£6,962

ICER = incremental cost effectiveness ratio; QALY = quality adjusted life year

*Algorithm interpretation

**Table 4:** Base Case 1: Incremental cost effectiveness analysis.

Strategy	Costs	QALYs	Incremental costs	Incremental QALYs	ICER/ QALY gained
Standard pathway	£514,187	447.963			
Kardia Mobile	£515,551	449.249	£1,364	1.286	£1,060
Generic lead-I device	£516,730	449.246	£1,179	-0.002	Dominated
RhythmPad GP*	£518,436	448.573	£2,885	-0.676	Dominated
Zenicor-ECG	£518,468	449.199	£2,917	-0.050	Dominated
MyDiagnostick	£521,233	449.024	£5,682	-0.225	Dominated
imPulse	£530,745	448.987	£15,194	-0.262	Dominated

ICER = incremental cost effectiveness ratio; QALY = quality adjusted life year

*Algorithm interpretation

The results of the pairwise analysis show that all lead-I ECG tests generated ICERs per QALY gained below the £20,000-£30,000 threshold usually considered to be cost effective by NICE. Kardia Mobile was the most cost effective option out of all the lead-I ECG tests included in the analysis, as it cost less and generated more benefits than each of the other devices. Scenario analyses were undertaken to investigate the impact on the ICER per QALY gained of varying some of the base case assumptions. Scenario analyses are presented in S1 Scenarios.

Each lead-I ECG test identified more AF cases than the standard diagnostic pathway. This came at a higher cost but with greater overall patient benefit in terms of mortality and quality of life. More patients were diagnosed following a lead-I ECG test due to the assumption that patients with paroxysmal AF would be in AF at the time of the initial consultation and that this would be captured by the lead-I ECG test. Some patients with paroxysmal AF would not be in AF at the time of a 12-lead ECG in the standard diagnostic pathway and so would remain undiagnosed. The extra cost of the lead-I pathway was primarily due to more patients receiving anticoagulant treatment, which was offset substantially but not entirely by a decrease in CVE rate (due to more patients with AF receiving treatment) and the associated lower cost of treating CVEs, particularly strokes. Lead-I ECGs were also associated with greater patient benefit in terms of lower mortality and higher quality of life as a result of experiencing fewer CVEs. This benefit may be reduced marginally by increased risk of clinically relevant bleeds due to more people receiving anticoagulant therapy. There was some extra cost and benefit associated with diagnosing patients more quickly than in the standard diagnostic pathway, but these were minimal.

## Discussion

No studies were identified that evaluated the diagnostic accuracy or the clinical impact of lead-I ECG devices in people presenting to primary care with signs or symptoms of AF and an irregular pulse. Consequently, the review focused on an asymptomatic population as pre-specified in the protocol.[4] We considered an asymptomatic population to comprise people not presenting with symptoms of AF, with or without a previous diagnosis of AF. These patients could have had co-existing cardiovascular conditions or could have been attending a cardiovascular clinic but did not present with signs or symptoms of AF. It is plausible that, if the population in the review had been people with signs or symptoms of AF and an irregular pulse, the sensitivity of lead-I ECG devices where the trace was interpreted by a trained healthcare professional would have been higher. However, it is also plausible that, in such a population, the specificity of lead-I ECG devices where the trace was interpreted by a trained healthcare professional would have been lower.

In the included studies, the sensitivity of lead-I ECG devices ranged from 80% to 100% and specificity ranged from 76% to 99% when the lead-I ECG trace was interpreted by a trained healthcare professional. The sensitivity results from the meta-analyses of lead-I ECG traces interpreted by a trained healthcare professional or lead-I ECG device algorithm (92%; 95% CI: 85% to 96%)[54] were similar to the sensitivity results reported for MPP in systematic reviews (91.6%; 95% CI: 75% to 98.6%).[17] The specificity values for lead-I ECG traces interpreted by a trained healthcare professional or lead-I ECG device algorithm were relatively higher (82%; 95% CI: 76% to 88%)[54] than those reported for MPP (78.8%; 95% CI: 51% to 94.5%).[17]

Lead-I ECG devices were more cost effective when there was a longer wait to 12-lead ECG and if the 12-lead ECG is performed in hospital. The majority of the patient benefit, however, comes after diagnosis due to a greater proportion of patients being correctly diagnosed with and treated for AF when compared to the standard diagnostic pathway, even if this benefit is slightly offset by an increased number of patients incorrectly diagnosed with AF with a lead-I ECG device. The proportion of patients with paroxysmal AF is uncertain in this population. If the proportion of patients with paroxysmal AF is lower than we assumed, lead-I ECG devices would be less cost effective compared with the standard diagnostic pathway.

In line with our findings, the results of recently published economic evaluations[17, 55] suggest that lead-I ECG devices may represent a cost effective use of resources for systematic, opportunistic screening of people aged 65 years and over during a routine GP appointment. Lead-I ECG devices may be cost effective for an asymptomatic population because only people that have a positive lead-I ECG test will have a subsequent 12-lead ECG test carried out. If a lead-I ECG test or an alternative screening test were not used, people with asymptomatic AF would remain undiagnosed until the time of an event (e.g., stroke). People with asymptomatic AF who are diagnosed early and receive appropriate treatment gain health benefits in comparison to people whose AF remains undiagnosed and who do not receive treatment for AF.

Currently, NICE recommends (CG180)[5] that an ECG is performed in all people (whether symptomatic or not) in whom AF is suspected because an irregular pulse has been detected. In updates to CG180[5] novel technologies to assist in the diagnosis of AF, such as lead-I ECG devices, need to be clearly distinguished from 12-lead ECG devices.

The main limitation of our study is that there are no published data evaluating the diagnostic accuracy, clinical impact or cost effectiveness of lead-I ECG devices for people presenting to primary care with signs or symptoms of AF and an irregular pulse. However, using diagnostic accuracy and clinical impact data from asymptomatic patients as a proxy, we present the results of the first economic evaluation of lead-I ECG devices for people presenting to primary care with signs or symptoms of AF and an irregular pulse; the economic evaluation considers the pathways for patients with signs or symptoms of AF and an irregular pulse presenting to the GP for an initial consultation.

## Conclusions

There is no evidence available for the use of single-time point lead-I ECG devices for the detection of AF in people with signs or symptoms of AF and an irregular pulse. The results of this assessment, using diagnostic accuracy data from asymptomatic patients as a proxy, suggest that lead-I ECG devices represent a cost effective use of NHS resources compared with MPP followed by a 12-lead ECG in primary or secondary care. The current standard pathway for the diagnosis of AF shows that patients with signs or symptoms of AF and an irregular pulse are advised to have a 12-lead ECG test. Given the assumptions in our model, the use of single-time point lead-I ECG devices in primary care for the detection of AF in people with signs or symptoms of AF and an irregular pulse appears to be a cost effective use of NHS resources compared with MPP followed by a 12-lead ECG.

## Supporting information

S1 Fig Diagnostic phase—Decision tree: Standard diagnostic pathway. (DOCX)

S2 Fig Diagnostic phase—Decision tree: Lead-I ECG diagnostic pathway (positive result). (DOCX)

S3 Fig Diagnostic phase—Decision tree: Lead-I ECG diagnostic pathway (negative result). (DOCX)

S4 Fig Diagnostic phase—Markov model. (DOCX)

S5 Fig Post-diagnostic phase—Markov model. (DOCX)

S6 Fig Summary receiver operating characteristic plots. (DOCX)

S1 Table PRISMA-DTA checklist. (DOCX)

S2 Table PRISMA-DTA for abstracts checklist. (DOCX)

S3 Table Eligibility criteria. (DOCX)

S4 Table Base case model assumptions. (DOCX)

S5 Table Cost per lead-I ECG test. (DOCX)

S6 Table Healthcare practitioner costs per 12-lead ECG test (primary and secondary care). (DOCX)

S7 Table Mortality rates and risk ratios (no previous CVEs) used in the economic model. (DOCX)

S8 Table Cardiovascular and adverse event rates. (DOCX)

S9 Table QUADAS-2 assessment of diagnostic test accuracy studies. (DOCX)

S10 Table Base case 1 costs, QALYs and patient outcomes. (DOCX)

S11 Table Results (base case 2 to 4). (DOCX)

S1 Text Search strategy (MEDLINE). (DOCX)

S2 Text Excluded studies. (DOCX)

S3 Text References that appear only in Supplementary Information. (DOCX)

S1 Scenarios Scenario analyses. (DOCX)
