# Supplementary material for: Lead-I ECG for detecting atrial fibrillation in patients attending primary care with an irregular pulse using single-time point testing: A systematic review and economic evaluation
Source: PLoS One. 2019 Dec 23;14(12):e0226671. doi: 10.1371/journal.pone.0226671 (PMC6927656; doi:10.1371/journal.pone.0226671)
Supplement: S5 Fig — (DOCX) [file pone.0226671.s005.docx]

## S5 Fig. Post-diagnostic phase - Markov model


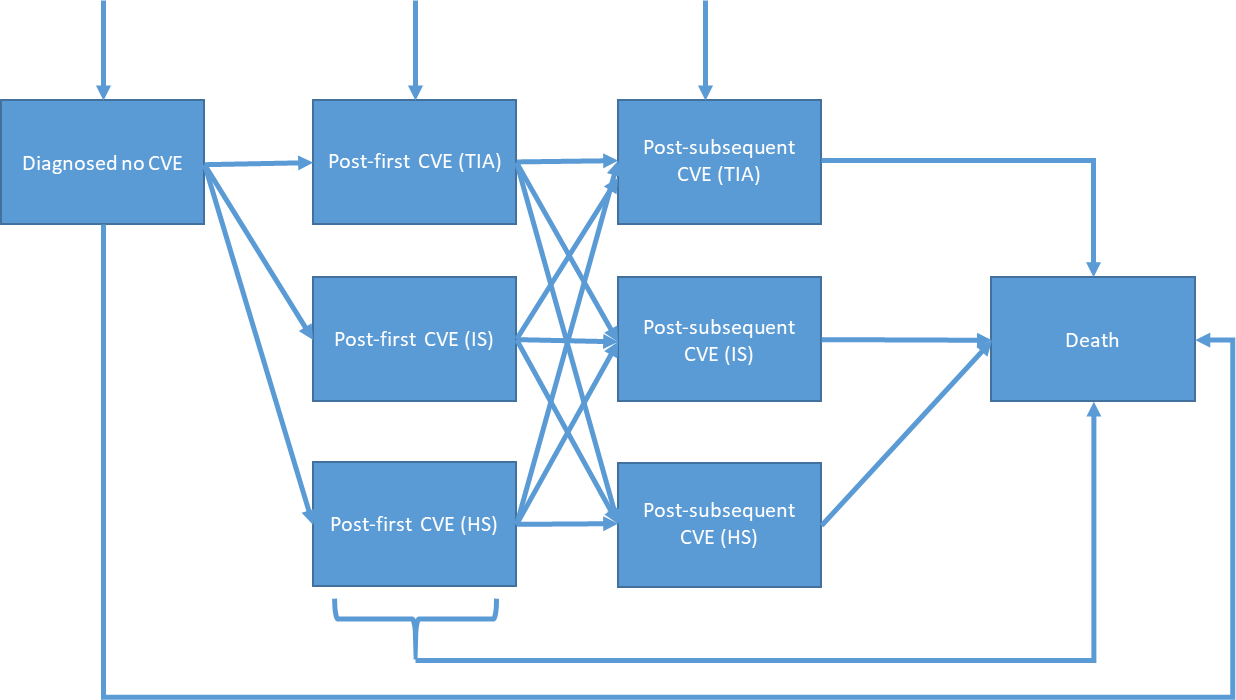


Note: death is possible from all states

CVE=cardiovascular event; HS=haemorrhagic stroke; IS=ischaemic stroke; TIA=transient ischaemic attack
