# Supplementary material for: Lead-I ECG for detecting atrial fibrillation in patients attending primary care with an irregular pulse using single-time point testing: A systematic review and economic evaluation
Source: PLoS One. 2019 Dec 23;14(12):e0226671. doi: 10.1371/journal.pone.0226671 (PMC6927656; doi:10.1371/journal.pone.0226671)
Supplement: S6 Fig — (DOCX) [file pone.0226671.s006.docx]

## S6 Fig. Summary receiver operating characteristic plots


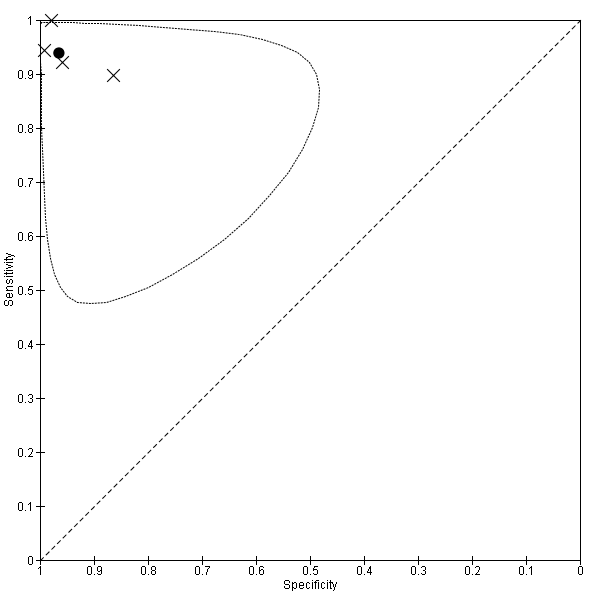


Figure A Summary receiver operating characteristic plot for lead-I ECG device as index test with trace interpreted by a trained healthcare professional and 12-lead ECG interpreted by a trained healthcare professional as reference standard (using Kardia Mobile lead-I ECG device and electrophysiologist 1 data from the Desteghe study)

X individual study result

meta-analysis result

confidence region


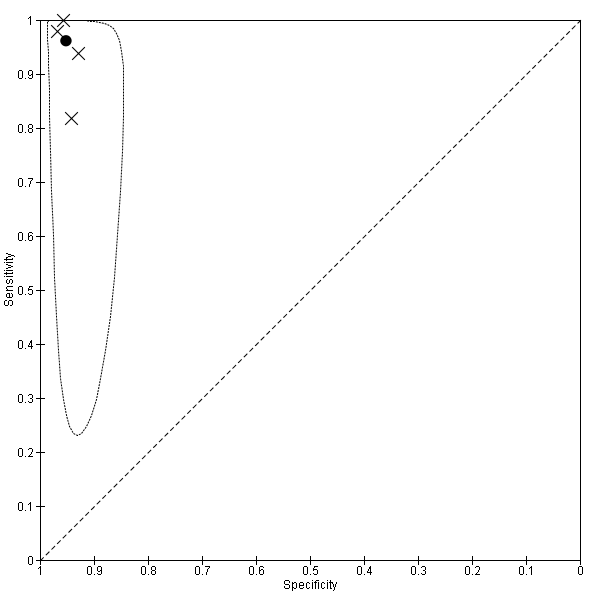


Figure B Summary receiver operating characteristic plot for lead-I ECG device as index test with trace interpreted by device algorithm and 12-lead ECG interpreted by a trained healthcare professional as reference standard (using MyDiagnostick lead-I ECG device data from the Desteghe study)

X individual study result

meta-analysis result

confidence region
