# Supplementary material for: Lead-I ECG for detecting atrial fibrillation in patients attending primary care with an irregular pulse using single-time point testing: A systematic review and economic evaluation
Source: PLoS One. 2019 Dec 23;14(12):e0226671. doi: 10.1371/journal.pone.0226671 (PMC6927656; doi:10.1371/journal.pone.0226671)
Supplement: S2 Table — (DOCX) [file pone.0226671.s008.docx]

## S2 Table. PRISMA-DTA for abstracts checklist

| **Section/topic** | **#** | **PRISMA-DTA for Abstracts Checklist item** | **Reported on page #** |
| --- | --- | --- | --- |
| **TITLE and PURPOSE** | | |  |
| Title | 1 | Identify the report as a systematic review (+/- meta-analysis) of diagnostic test accuracy (DTA) studies. | p 1 |
| Objectives | 2 | Indicate the research question, including components such as participants, index test, and target conditions. | p 2 |
| **METHODS** | | |  |
| Eligibility criteria | 3 | Include study characteristics used as criteria for eligibility. | p 2 |
| Information sources | 4 | List the key databases searched and the search dates. | p 2 |
| Risk of bias & applicability | 5 | Indicate the methods of assessing risk of bias and applicability. | NR |
| Synthesis of results | A1 | Indicate the methods for the data synthesis. | NR |
| **RESULTS** | | |  |
| Included studies | 6 | Indicate the number and type of included studies and the participants and relevant characteristics of the studies (including the reference standard). | p 2 |
| Synthesis of results | 7 | Include the results for the analysis of diagnostic accuracy, preferably indicating the number of studies and participants. Describe test accuracy including variability; if meta-analysis was done, include summary results and confidence intervals. | p 2 |
| **DISCUSSION** | | |  |
| Strengths and limitations | 9 | Provide a brief summary of the strengths and limitations of the evidence | p 3 |
| Interpretation | 10 | Provide a general interpretation of the results and the important implications. | p 3 |
| **OTHER** | | |  |
| Funding | 11 | Indicate the primary source of funding for the review. | p 3 |
| Registration | 12 | Provide the registration number and the registry name | p 3 |
