# Supplementary material for: Lead-I ECG for detecting atrial fibrillation in patients attending primary care with an irregular pulse using single-time point testing: A systematic review and economic evaluation
Source: PLoS One. 2019 Dec 23;14(12):e0226671. doi: 10.1371/journal.pone.0226671 (PMC6927656; doi:10.1371/journal.pone.0226671)
Supplement: S3 Table — (DOCX) [file pone.0226671.s009.docx]

## S3 Table. Eligibility criteria

| Population | (1) People with signs or symptoms that may indicate underlying AF and who have an irregular pulse; (2) Asymptomatic population* if no evidence for (1) is available | |
| --- | --- | --- |
| Setting | Primary care (ideal), secondary or tertiary care | |
| Index tests | Lead-I ECG using one of the following technologies:   - imPulse - Kardia Mobile - MyDiagnostick - RhythmPad GP - Zenicor-ECG | |
|  | **Clinical impact** | **Diagnostic test accuracy** |
| Comparator | Manual pulse palpation followed by a 12-lead ECG in primary or secondary care prior to initiation of anticoagulation therapy or other lead-I ECG devices as specified above | Other lead-I ECG devices as specified above, or no comparator |
| Reference standard | Not applicable | 12-lead ECG performed and interpreted by a trained healthcare professional |
| Outcomes (at least one) | Intermediate outcomes   - Time to diagnosis of AF - Time to initiation of preventative treatment (such as interventions to prevent stroke) - Concordance between lead-I ECG devices - Test failure rate - Time to complete testing and store produced ECG trace - Ease of use of devices (for patients and healthcare professionals), including training requirements - Impact of test results on clinical decision making - Number of 12-lead ECGs carried out - Diagnostic yield (number of AF diagnoses) | Diagnostic test accuracy   - Numbers of true positive, false negative, false positive and true negative test results |
|  | Clinical outcomes   - Mortality - Morbidity (including stroke, other thromboembolisms and heart failure, and any complications arising from preventative treatments, such as adverse effects of anti-arrhythmic, rate control or anticoagulation treatment) |  |
|  | Patient-reported outcomes   - Health-related quality of life - Acceptability of the devices |  |
| Study design | RCTs, cross-sectional, case-control, cohort studies and uncontrolled single arm studies. Qualitative studies were considered to evaluate the ease of use of the devices | Diagnostic cross-sectional and case-control studies |

AF=atrial fibrillation; ECG=electrocardiogram; RCT=randomised controlled trial

* Asymptomatic population defined as people presenting with no symptoms of AF, with or without previously diagnosed AF
