# Supplementary material for: Lead-I ECG for detecting atrial fibrillation in patients attending primary care with an irregular pulse using single-time point testing: A systematic review and economic evaluation
Source: PLoS One. 2019 Dec 23;14(12):e0226671. doi: 10.1371/journal.pone.0226671 (PMC6927656; doi:10.1371/journal.pone.0226671)
Supplement: S4 Table — (DOCX) [file pone.0226671.s010.docx]

## S4 Table. Base case model assumptions

| Parameter | Assumption or source | Justification |
| --- | --- | --- |
| AF status at initial consultation | All patients with AF are in AF at the time of the initial consultation | Population is patients presenting to primary with signs or symptoms of AF and an irregular pulse. These symptoms are assumed to be caused by AF if the patient has AF |
| Mean age | 70 years | Mean age observed in RCTs used by Sterne^56^ and to estimate CVE rate parameters |
| % female | 51.6% | Age-adjusted proportion in the general population,^57^ assumed to match proportion in GP lists |
| AF prevalence | Male: 6.7%  Female: 3.4% | Recent data from UK primary care^15^ |
| Proportion of AF undiagnosed | 13.1% | Recent data ^58^ |
| Proportion of AF with signs or symptoms | Male: 57.5%  Female: 67.9% | Recent data ^16^ |
| Proportion of patients with undiagnosed symptomatic AF who have paroxysmal AF | 50% | Assumption due to wide range reported by Welton and the lack of evidence specifically on incidence rates for symptomatic paroxysmal AF |
| Number of lead-I ECG devices per practice | One per GP | Previous economic evaluation^17^ |
| Proportion of lead-I ECG tests reviewed by cardiologist | 10% | Assumption (used in Welton et al^17^ on the basis of advice from clinical experts) |
| Extra time taken to administer lead-I ECG test | 0 minutes | Test is assumed to be administered during standard GP appointment |
| Proportion of patients receiving anticoagulation | 66.8% | Calculated from proportion of AF patients with CHA2DS2-VASc ≥2 and proportion of these patients who receive anticoagulation^21^ |
| Proportion of patients receiving anticoagulation who receive NOACs | 100% | Simplifying assumption based on evidence that prescriptions for NOACs overtook prescriptions for warfarin in 2018 |
| Time from diagnosis to anticoagulation | Immediate | Simplifying assumption allowing the maximum potential benefit from earlier diagnosis with lead-I ECG |
| Proportion of patients receiving 12-lead ECG | 100% for standard pathway and lead-I positive  80% for lead-I negative | Standard pathway: NICE CG180^5^  Lead-I positive (AF diagnosed): NICE CG180^5^  Lead-I negative: assumption based on clinical advice (Appendix 11) and varied in sensitivity analyses |
| Diagnostic accuracy of 12-lead ECG | 100% sensitivity and specificity for those patients in AF at time of test | 12-lead ECG is reference test for lead-I devices, hence must be assumed to be 100% accurate |
| Proportion of patients with paroxysmal AF not in AF at time of 12-lead ECG | 47.5% | Data from Israel 2004^59^. Calculated as 46 patients who did not have AF documented by resting ECG out of 97 patients with device detected AF during follow up |
| Diagnostic accuracy of Holter monitor | 100% sensitivity and specificity for those patients in AF at time of test | Simplifying assumption |
| Proportion of patients with paroxysmal AF not in AF at time of Holter monitor | 30% | Data from Kirchoff 2006^60^ |
