# Supplementary material for: Lead-I ECG for detecting atrial fibrillation in patients attending primary care with an irregular pulse using single-time point testing: A systematic review and economic evaluation
Source: PLoS One. 2019 Dec 23;14(12):e0226671. doi: 10.1371/journal.pone.0226671 (PMC6927656; doi:10.1371/journal.pone.0226671)
Supplement: S5 Table — (DOCX) [file pone.0226671.s011.docx]

## S5 Table. Cost per lead-I ECG test

Table A Cost per lead-I ECG test

| Device | Annual device cost (exc. VAT) | Number of patients tested per year | Peripherals cost per test | Unit cost per test* |
| --- | --- | --- | --- | --- |
| imPulse | £87.50 | 54 | 0.00 | £1.62 |
| Kardia Mobile | £16.50 | 54 | 0.00 | £0.31 |
| MyDiagnostick | £90.00 | 54 | 0.00 | £1.67 |
| RhythmPad GP | £1,100.00 | 54 | 0.00 | £20.42 |
| Zenicor ECG | £613.27 | 54 | 0.02 | £11.40 |
| Generic lead-I device | £381.45 | 54 | 0.02 | £7.10 |

*Some costs may not calculate precisely due to rounding

Table B Cost per administration and interpretation of lead-I ECG test (base case)

|  | Unit cost | Source | Time taken | Cost per test |
| --- | --- | --- | --- | --- |
| Algorithm | £0 |  | 0 | £0 |
| GP | £0 |  | 0 | £0 |
| Cardiologist | £107 per hour | PSSRU^62^ | 1 minute* | £1.78 |

*Based on data from Hobbs^61^
