# Supplementary material for: Lead-I ECG for detecting atrial fibrillation in patients attending primary care with an irregular pulse using single-time point testing: A systematic review and economic evaluation
Source: PLoS One. 2019 Dec 23;14(12):e0226671. doi: 10.1371/journal.pone.0226671 (PMC6927656; doi:10.1371/journal.pone.0226671)
Supplement: S6 Table — (DOCX) [file pone.0226671.s012.docx]

## S6 Table. Healthcare practitioner costs per 12-lead ECG test (primary and secondary care)

|  | Unit cost | Source | Activity | Time taken | Cost per test |
| --- | --- | --- | --- | --- | --- |
| Primary care | | | | | |
| Device | £2.25 per use | Estimate |  |  | £2.25 |
| Disposables | £1.13 per use | Hobbs^61^ |  |  | £1.13 |
| Nurse | £42 per hour | PSSRU^62^ | Administration | 7 minutes* | £4.90 |
| GP | £137 per hour | PSSRU^62^ | Interpretation | 1 minute* | £2.28 |
| Cardiologist | £107 per hour | PSSRU^62^ | Interpretation | 1 minute* | £1.78 |
| Total cost per 12-lead ECG test in primary care | | | | | £12.34 |
| Secondary care | | | | | |
| Electrocardiogram Monitoring or Stress Testing | £52 per test | NHS Reference costs 2016/17 (HRG: EY51Z DADS)^63^ |  | N/A | £52 |

HRG=Healthcare Resource Group; DADS=directly accessed diagnostic services

*Based on data from Hobbs^61^
