# Supplementary material for: Lead-I ECG for detecting atrial fibrillation in patients attending primary care with an irregular pulse using single-time point testing: A systematic review and economic evaluation
Source: PLoS One. 2019 Dec 23;14(12):e0226671. doi: 10.1371/journal.pone.0226671 (PMC6927656; doi:10.1371/journal.pone.0226671)
Supplement: S7 Table — (DOCX) [file pone.0226671.s013.docx]

## S7 Table. Mortality rates and risk ratios (no previous CVEs) used in the economic model

| State | Source | Value type | Value | Use |
| --- | --- | --- | --- | --- |
| AF: treated: NOAC | Sterne 2017^56^ | HR versus warfarin (under 80 years) | 0.89 |  |
| AF: untreated | Sterne 2017^56^ | HR versus warfarin | 1.178 |  |
| AF: treated: Warfarin | Sterne 2017^56^ | Annual rate (70 years) | 0.038 | Reference value |
| No AF: treated: NOAC  No AF: untreated | ONS^57^ | Annual rate | Various |  |

ONS=Office of National Statistics; HR=hazard ratio; AF=atrial fibrillation; HR=hazard ratio; NOAC=new oral anticoagulant
