# Supplementary material for: Lead-I ECG for detecting atrial fibrillation in patients attending primary care with an irregular pulse using single-time point testing: A systematic review and economic evaluation
Source: PLoS One. 2019 Dec 23;14(12):e0226671. doi: 10.1371/journal.pone.0226671 (PMC6927656; doi:10.1371/journal.pone.0226671)
Supplement: S8 Table — (DOCX) [file pone.0226671.s014.docx]

## S8 Table. Cardiovascular and adverse event rates

Table A Cardiovascular and adverse event rates: ischaemic stroke

| State | Source | Value type | Value |
| --- | --- | --- | --- |
| AF: treated: NOAC | Sterne 2017^56^ | HR versus warfarin | 0.9 |
| AF: treated: warfarin | Sterne 2017^56^ | Annual rate (70 years) | 0.012 |
| AF: untreated | Sterne 2017^56^ | HR versus warfarin | 1.178 |
| No AF: treated: NOAC |  |  | General population |
| No AF: untreated | PHE 2018^64^ | Annual rate (female, 70 years) | 0.0030 |
|  |  | Annual rate (female, 80 years) | 0.0060 |
|  |  | Annual rate (female, 90 years) | 0.0108 |
|  |  | Annual rate (male, 70 years) | 0.0044 |
|  |  | Annual rate (male, 80 years) | 0.0064 |
|  |  | Annual rate (male, 90 years) | 0.0099 |

PHE=Public Health England; HR=hazard ratio; AF=atrial fibrillation; NOAC=new oral anticoagulants

Table B Cardiovascular and adverse event rates: bleed

| State | Source | Value type | Value |
| --- | --- | --- | --- |
| AF: treated: NOAC | Sterne 2017^56^ | HR versus warfarin | 0.82 |
| AF: treated: warfarin | Sterne 2017^56^ | Annual rate (70 years) | 0.066 |
| AF: untreated | Sterne 2017^56^ | HR versus warfarin | 0.543 |
| No AF: treated: NOAC | Calculated | HR versus untreated | 1.511 |
| No AF: untreated | NHS Reference Costs 2016/17^63^  Includes: gastrointestinal bleed (FD03A:FD03H),  unspecified haematuria (LB38C:LB38H),  non-malignant GI tract disorders (FD10A: FD10M) | Annual rate (assume 70 years) | 0.011* |

HR=hazard ratio; AF=atrial fibrillation; NOAC=new oral anticoagulants

*Estimated as incidence of activity reported in NHS Reference Costs^63^ per population in England (19 or over) reported by the Office for National Statistics^57^

Table C Cardiovascular and adverse event rates: transient ischaemic attack

| State | Source | Value type | Value |
| --- | --- | --- | --- |
| AF: treated: NOAC | Sterne 2017^56^ | HR versus warfarin | 0.740 |
| AF: treated: Warfarin | Sterne 2017^56^ | Annual rate (70 years) | 0.025 |
| AF: untreated | Sterne 2017^56^ | HR versus warfarin | 1.617 |
| No AF: treated: NOAC |  |  | General population |
| No AF: untreated | Rothwell 2005^65^* | Annual rate (female, 70 years) | 0.0022 |
|  |  | Annual rate (female, 80 years) | 0.0057 |
|  |  | Annual rate (female, 90 years) | 0.0093 |
|  |  | Annual rate (male, 70 years) | 0.0014 |
|  |  | Annual rate (male, 80 years) | 0.0034 |
|  |  | Annual rate (male, 90 years) | 0.0080 |

HR=hazard ratio; AF=atrial fibrillation; NOAC=new oral anticoagulants

*Incidence rates estimated from published figures

Table D Cardiovascular and adverse event rates: haemorrhagic stroke

| State | Source | Value type | Value |
| --- | --- | --- | --- |
| AF: treated: NOAC | Sterne 2017^56^ | HR versus warfarin | 0.46 |
| AF: treated: warfarin | Sterne 2017^56^ | Annual rate (70 years) | 0.009 |
| AF: untreated | Sterne 2017^56^ | HR versus warfarin | 0.543 |
| No AF: treated: NOAC |  |  | General population |
| No AF: untreated | Rothwell 2005^65^* | Annual rate (female, 70 years) | 0.00034 |
|  |  | Annual rate (female, 80 years) | 0.00100 |
|  |  | Annual rate (female, 90 years) | 0.00104 |
|  |  | Annual rate (male, 70 years) | 0.00026 |
|  |  | Annual rate (male, 80 years) | 0.00171 |
|  |  | Annual rate (male, 90 years) | 0.00078 |

HR=hazard ratio; AF=atrial fibrillation; NOAC=new oral anticoagulants

*Incidence rates estimated from published figures

Table E Probability of subsequent stroke and the proportion of subsequent strokes that are TIA, IS or HS

| Event | | Base case | Source |
| --- | --- | --- | --- |
|  |  |  |  |
| Probability of subsequent CVE (annual) | Year 1 | 0.065 | Mohan 2011^66^ |
|  | Year 2 onwards | 0.038 |  |
| Probability that subsequent CVE is: | TIA | 0.640 | Rothwell 2005^65^ |
|  | IS | 0.057 |  |
|  | HS | 0.303 |  |

CVE=cardiovascular event; HS=haemorrhagic stroke; IS=ischaemic stroke; TIA=transient ischaemic attack
