# Supplementary material for: Lead-I ECG for detecting atrial fibrillation in patients attending primary care with an irregular pulse using single-time point testing: A systematic review and economic evaluation
Source: PLoS One. 2019 Dec 23;14(12):e0226671. doi: 10.1371/journal.pone.0226671 (PMC6927656; doi:10.1371/journal.pone.0226671)
Supplement: S9 Table — (DOCX) [file pone.0226671.s015.docx]

## S9 Table. QUADAS-2 assessment of diagnostic test accuracy studies

| Study | Risk of bias | | | | Applicability concerns | | |
| --- | --- | --- | --- | --- | --- | --- | --- |
|  | Patient selection | Index test | Reference standard | Flow and timing | Patient selection | Index test | Reference standard |
| * Crockford 2013^37^ | Unclear | Unclear | Unclear | Unclear | High | Unclear | Low |
| Desteghe 2017^28^ | Unclear | Low | Low | Low | High | Low | Low |
| Doliwa 2009^29^ | Unclear | Low | Low | Low | High | Low | Low |
| Haberman 2015^31^ | Unclear | Unclear | Unclear | Low | High | Low | Low |
| ** Koltowski 2017^38^ | Unclear | Unclear | Unclear | Low | High | Unclear | Low |
| Lau 2013^33^ | Unclear | Low | Low | Low | High | High | Low |
| Tieleman 2014^34^ | Unclear | Low | Low | Low | High | High | Low |
| Vaes 2014^35^ | Unclear | Low | Low | Unclear | High | High | Low |
| Williams 2015^36^ | Unclear | Low | Low | Unclear | High | Low | Low |

*The poster based on the conference proceeding by Crockford^37^ was provided and used for the purposes of data extraction and quality assessment

**The study by Koltowski^38^ was available only as a conference proceeding
