# Supplementary material for: Lead-I ECG for detecting atrial fibrillation in patients attending primary care with an irregular pulse using single-time point testing: A systematic review and economic evaluation
Source: PLoS One. 2019 Dec 23;14(12):e0226671. doi: 10.1371/journal.pone.0226671 (PMC6927656; doi:10.1371/journal.pone.0226671)
Supplement: S10 Table — (DOCX) [file pone.0226671.s016.docx]

## S10 Table. Base case 1 costs, QALYs and patient outcomes

Table A Base Case 1: Total costs of annual number of symptomatic patients with positive MPP seen by a single GP’

| Strategy | Lead-I ECG test | Treatment (NOACs & rate control) | CVEs and AEs | 12-lead ECG | Paroxysmal testing (holter monitor) | Total costs |
| --- | --- | --- | --- | --- | --- | --- |
| Standard pathway | £0 | £90,630 | £420,279 | £536 | £2,743 | £514,187 |
| Kardia Mobile | £26 | £102,952 | £409,881 | £452 | £2,240 | £515,551 |
| imPulse | £97 | £116,317 | £411,612 | £454 | £2,265 | £530,745 |
| MyDiagnostick | £100 | £107,077 | £411,358 | £451 | £2,247 | £521,233 |
| Generic lead-I device | £392 | £103,746 | £409,898 | £452 | £2,242 | £516,730 |
| Zenicor-ECG | £624 | £104,938 | £410,210 | £452 | £2,244 | £518,468 |
| RhythmPad GP* | £1,110 | £100,358 | £414,292 | £446 | £2,231 | £518,436 |

AE=adverse events; CVE=cardiovascular events

*Algorithm interpretation

Table B Base Case 1: QALYs and patient outcomes

| Strategy | IS | **HS** | **TIA** | **False negatives** | **False positives** | Bleeds | Total QALYs |
| --- | --- | --- | --- | --- | --- | --- | --- |
| Standard pathway | 11.621 | 2.124 | 8.406 | 1.606 | 0.000 | 23.581 | 447.963 |
| Kardia Mobile | 11.452 | 1.996 | 8.359 | 0.144 | 1.379 | 23.751 | 449.249 |
| imPulse | 11.482 | 2.019 | 8.366 | 0.397 | 3.663 | 23.730 | 448.987 |
| MyDiagnostick | 11.478 | 2.015 | 8.365 | 0.361 | 2.155 | 23.720 | 449.024 |
| Generic lead-I device | 11.452 | 1.996 | 8.359 | 0.147 | 1.508 | 23.752 | 449.246 |
| Zenicor-ECG | 11.457 | 2.000 | 8.360 | 0.193 | 1.724 | 23.746 | 449.199 |
| RhythmPad GP* | 11.530 | 2.054 | 8.377 | 0.794 | 1.293 | 23.630 | 448.573 |

AE=adverse events; CVE=cardiovascular events; QALY=quality adjusted life year; IS=ischaemic stroke; HS=haemhorragic stroke; TIA=transient ischaemic accident

*Algorithm interpretation
