# Supplementary material for: Lead-I ECG for detecting atrial fibrillation in patients attending primary care with an irregular pulse using single-time point testing: A systematic review and economic evaluation
Source: PLoS One. 2019 Dec 23;14(12):e0226671. doi: 10.1371/journal.pone.0226671 (PMC6927656; doi:10.1371/journal.pone.0226671)
Supplement: S11 Table — (DOCX) [file pone.0226671.s017.docx]

## S11 Table. Results (base case 2 to 4)

Base Case 2: 12-lead ECG in primary care, 14 days to 12-lead ECG

Table A Base Case 2: Total costs of annual number of symptomatic patients with positive MPP seen by a single GP

| Strategy | Lead-I ECG test | Treatment (NOACs & rate control) | CVEs and AEs | 12-lead ECG | Paroxysmal testing (holter monitor) | Total costs |
| --- | --- | --- | --- | --- | --- | --- |
| Standard pathway | £0 | £90,431 | £420,710 | £535 | £2,741 | £514,416 |
| Kardia Mobile | £26 | £102,842 | £409,851 | £451 | £2,239 | £515,408 |
| imPulse | £97 | £116,189 | £411,588 | £453 | £2,263 | £530,590 |
| MyDiagnostick | £100 | £106,951 | £411,334 | £451 | £2,245 | £521,080 |
| Generic lead-I device | £392 | £103,636 | £409,868 | £451 | £2,240 | £516,587 |
| Zenicor-ECG | £624 | £104,824 | £410,181 | £451 | £2,242 | £518,323 |
| RhythmPad GP* | £1,110 | £100,198 | £414,279 | £445 | £2,229 | £518,261 |

AE=adverse events; CVE=cardiovascular events

*Algorithm interpretation

Table B Base Case 2: QALYs and patient outcomes

| Strategy | IS | **HS** | **TIA** | **False negatives** | **False positives** | Bleeds | Total QALYs |
| --- | --- | --- | --- | --- | --- | --- | --- |
| Standard pathway | 11.620 | 2.123 | 8.407 | 1.606 | 0.000 | 23.572 | 447.895 |
| Kardia Mobile | 11.451 | 1.996 | 8.358 | 0.144 | 1.378 | 23.743 | 449.220 |
| imPulse | 11.482 | 2.018 | 8.365 | 0.396 | 3.660 | 23.721 | 448.956 |
| MyDiagnostick | 11.477 | 2.015 | 8.364 | 0.360 | 2.153 | 23.711 | 448.994 |
| Generic lead-I device | 11.451 | 1.996 | 8.358 | 0.147 | 1.507 | 23.744 | 449.217 |
| Zenicor-ECG | 11.457 | 2.000 | 8.360 | 0.192 | 1.722 | 23.738 | 449.170 |
| RhythmPad GP* | 11.529 | 2.054 | 8.376 | 0.793 | 1.292 | 23.620 | 448.540 |

AE=adverse events; CVE=cardiovascular events; QALY=quality adjusted life year; IS=ischaemic stroke; HS=haemhorragic stroke; TIA=transient ischaemic accident

*Algorithm interpretation

Table C Base Case 2: Pairwise cost effectiveness analysis

| Strategy | Costs | QALYs | Incremental costs | Incremental QALYs | ICER/ QALY gained |
| --- | --- | --- | --- | --- | --- |
| Standard pathway | £514,416 | 447.895 |  |  |  |
| Kardia Mobile | £515,408 | 449.220 | £1,221 | 1.257 | £971 |
| imPulse | £530,590 | 448.956 | £16,403 | 0.994 | £16,506 |
| MyDiagnostick | £521,080 | 448.994 | £6,892 | 1.031 | £6,684 |
| Generic lead-I device | £516,587 | 449.217 | £2,400 | 1.255 | £1,912 |
| Zenicor-ECG | £518,323 | 449.170 | £4,135 | 1.207 | £3,426 |
| RhythmPad GP* | £518,261 | 448.540 | £4,073 | 0.577 | £7,055 |

ICER=incremental cost effectiveness ratio; QALY=quality adjusted life year

*Algorithm interpretation

Table D Base Case 2: Incremental cost effectiveness analysis

| Strategy | Costs | QALYs | Incremental costs | Incremental QALYs | ICER/ QALY gained |
| --- | --- | --- | --- | --- | --- |
| Standard pathway | £514,416 | 447.895 |  |  |  |
| Kardia Mobile | £515,408 | 449.220 | £992 | 1.324 | £749 |
| Generic lead-I device | £516,587 | 449.217 | £1,179 | -0.002 | Dominated |
| RhythmPad GP* | £518,261 | 448.540 | £2,853 | -0.680 | Dominated |
| Zenicor-ECG | £518,323 | 449.170 | £2,915 | -0.050 | Dominated |
| MyDiagnostick | £521,080 | 448.994 | £5,672 | -0.226 | Dominated |
| imPulse | £530,590 | 448.956 | £15,182 | -0.264 | Dominated |

ICER=incremental cost effectiveness ratio; QALY=quality adjusted life year

*Algorithm interpretation

Base Case 3: 12-lead ECG in secondary care, 2 days to 12-lead ECG

Table E Base Case 3: Total costs of annual number of symptomatic patients with positive MPP seen by a single GP

| Strategy | Lead-I ECG test | Treatment (NOACs & rate control) | CVEs and AEs | 12-lead ECG | Paroxysmal testing (holter monitor) | Total costs |
| --- | --- | --- | --- | --- | --- | --- |
| Standard pathway | £0 | £90,630 | £420,279 | £2,801 | £2,743 | £516,453 |
| Kardia Mobile | £26 | £102,952 | £409,881 | £2,361 | £2,240 | £517,460 |
| imPulse | £97 | £116,317 | £411,612 | £2,373 | £2,265 | £532,663 |
| MyDiagnostick | £100 | £107,077 | £411,358 | £2,359 | £2,247 | £523,140 |
| Generic lead-I device | £392 | £103,746 | £409,898 | £2,362 | £2,242 | £518,640 |
| Zenicor-ECG | £624 | £104,938 | £410,210 | £2,362 | £2,244 | £520,378 |
| RhythmPad GP* | £1,110 | £100,358 | £414,292 | £2,330 | £2,231 | £520,320 |

AE=adverse events; CVE=cardiovascular events

*Algorithm interpretation

Table F Base Case 3: QALYs and patient outcomes

| Strategy | IS | **HS** | **TIA** | **False negatives** | **False positives** | Bleeds | Total QALYs |
| --- | --- | --- | --- | --- | --- | --- | --- |
| Standard pathway | 11.621 | 2.124 | 8.406 | 1.606 | 0.000 | 23.581 | 447.963 |
| Kardia Mobile | 11.452 | 1.996 | 8.359 | 0.144 | 1.379 | 23.751 | 449.249 |
| imPulse | 11.482 | 2.019 | 8.366 | 0.397 | 3.663 | 23.730 | 448.987 |
| MyDiagnostick | 11.478 | 2.015 | 8.365 | 0.361 | 2.155 | 23.720 | 449.024 |
| Generic lead-I device | 11.452 | 1.996 | 8.359 | 0.147 | 1.508 | 23.752 | 449.246 |
| Zenicor-ECG | 11.457 | 2.000 | 8.360 | 0.193 | 1.724 | 23.746 | 449.199 |
| RhythmPad GP* | 11.530 | 2.054 | 8.377 | 0.794 | 1.293 | 23.630 | 448.573 |

AE=adverse events; CVE=cardiovascular events; QALY=quality adjusted life year; IS=ischaemic stroke; HS=haemhorragic stroke; TIA=transient ischaemic accident

*Algorithm interpretation

Table G Base Case 3: Pairwise cost effectiveness analysis

| Strategy | Costs | QALYs | Incremental costs | Incremental QALYs | ICER/ QALY gained |
| --- | --- | --- | --- | --- | --- |
| Standard pathway | £516,453 | 447.963 |  |  |  |
| Kardia Mobile | £517,460 | 449.249 | £3,273 | 1.286 | £2,544 |
| imPulse | £532,663 | 448.987 | £18,476 | 1.024 | £18,038 |
| MyDiagnostick | £523,140 | 449.024 | £8,953 | 1.061 | £8,435 |
| Generic lead-I device | £518,640 | 449.246 | £4,453 | 1.284 | £3,468 |
| Zenicor-ECG | £520,378 | 449.199 | £6,191 | 1.236 | £5,007 |
| RhythmPad GP* | £520,320 | 448.573 | £6,133 | 0.610 | £10,048 |

ICER=incremental cost effectiveness ratio; QALY=quality adjusted life year

*Algorithm interpretation

Table H Base Case 3: Incremental cost effectiveness analysis

| Strategy | Costs | QALYs | Incremental costs | Incremental QALYs | ICER/ QALY gained |
| --- | --- | --- | --- | --- | --- |
| Standard pathway | £516,453 | 447.963 |  |  |  |
| Kardia Mobile | £517,460 | 449.249 | £1,007 | 1.286 | £783 |
| imPulse | £518,640 | 449.246 | £1,180 | -0.002 | Dominated |
| MyDiagnostick | £520,320 | 448.573 | £2,860 | -0.676 | Dominated |
| Generic lead-I device | £520,378 | 449.199 | £2,918 | -0.050 | Dominated |
| Zenicor-ECG | £523,140 | 449.024 | £5,680 | -0.225 | Dominated |
| RhythmPad GP* | £532,663 | 448.987 | £15,203 | -0.262 | Dominated |

ICER=incremental cost effectiveness ratio; QALY=quality adjusted life year

*Algorithm interpretation

Base Case 4: 12-lead ECG in secondary care, 14 days to 12-lead ECG

Table I Base Case 4: Total costs of annual number of symptomatic patients with positive MPP seen by a single GP

| Strategy | Lead-I ECG test | Treatment (NOACs & rate control) | CVEs and AEs | 12-lead ECG | Paroxysmal testing (holter monitor) | Total costs |
| --- | --- | --- | --- | --- | --- | --- |
| Standard pathway | £0 | £90,431 | £420,710 | £2,797 | £2,741 | £516,678 |
| Kardia Mobile | £26 | £102,842 | £409,851 | £2,358 | £2,239 | £517,315 |
| imPulse | £97 | £116,189 | £411,588 | £2,370 | £2,263 | £532,507 |
| MyDiagnostick | £100 | £106,951 | £411,334 | £2,356 | £2,245 | £522,985 |
| Generic lead-I device | £392 | £103,636 | £409,868 | £2,359 | £2,240 | £518,495 |
| Zenicor-ECG | £624 | £104,824 | £410,181 | £2,359 | £2,242 | £520,231 |
| RhythmPad GP* | £1,110 | £100,198 | £414,279 | £2,327 | £2,229 | £520,142 |

AE=adverse events; CVE=cardiovascular events

*Algorithm interpretation

Table J Base Case 4: QALYs and patient outcomes

| Strategy | IS | **HS** | **TIA** | **False negatives** | **False positives** | Bleeds | Total QALYs |
| --- | --- | --- | --- | --- | --- | --- | --- |
| Standard pathway | 11.620 | 2.123 | 8.407 | 1.606 | 0.000 | 23.572 | 447.895 |
| Kardia Mobile | 11.451 | 1.996 | 8.358 | 0.144 | 1.378 | 23.743 | 449.220 |
| imPulse | 11.482 | 2.018 | 8.365 | 0.396 | 3.660 | 23.721 | 448.956 |
| MyDiagnostick | 11.477 | 2.015 | 8.364 | 0.360 | 2.153 | 23.711 | 448.994 |
| Generic lead-I device | 11.451 | 1.996 | 8.358 | 0.147 | 1.507 | 23.744 | 449.217 |
| Zenicor-ECG | 11.457 | 2.000 | 8.360 | 0.192 | 1.722 | 23.738 | 449.170 |
| RhythmPad GP* | 11.529 | 2.054 | 8.376 | 0.793 | 1.292 | 23.620 | 448.540 |

AE=adverse events; CVE=cardiovascular events; QALY=quality adjusted life year; IS=ischaemic stroke; HS=haemhorragic stroke; TIA=transient ischaemic accident

*Algorithm interpretation

Table K Base Case 4: Pairwise cost effectiveness analysis

| Strategy | Costs | QALYs | Incremental Costs | Incremental QALYs | ICER/ QALY gained |
| --- | --- | --- | --- | --- | --- |
| Standard pathway | £516,678 | 447.895 |  |  |  |
| Kardia Mobile | £517,315 | 449.220 | £3,127 | 1.257 | £2,487 |
| imPulse | £532,507 | 448.956 | £18,319 | 0.994 | £18,435 |
| MyDiagnostick | £522,985 | 448.994 | £8,797 | 1.031 | £8,532 |
| Generic lead-I device | £518,495 | 449.217 | £4,307 | 1.255 | £3,433 |
| Zenicor-ECG | £520,231 | 449.170 | £6,043 | 1.207 | £5,006 |
| RhythmPad GP* | £520,142 | 448.540 | £5,955 | 0.577 | £10,314 |

ICER=incremental cost effectiveness ratio; QALY=quality adjusted life year

*Algorithm interpretation

Table L Base Case 4: Incremental cost effectiveness analysis

| Strategy | Costs | QALYs | Incremental Costs | Incremental QALYs | ICER/ QALY gained |
| --- | --- | --- | --- | --- | --- |
| Standard pathway | £516,678 | 447.895 |  |  |  |
| Kardia Mobile | £517,315 | 449.220 | £637 | 1.324 | £481 |
| Generic lead-I device | £518,495 | 449.217 | £1,180 | -0.002 | Dominated |
| RhythmPad GP* | £520,142 | 448.540 | £2,828 | -0.680 | Dominated |
| Zenicor-ECG | £520,231 | 449.170 | £2,916 | -0.050 | Dominated |
| MyDiagnostick | £522,985 | 448.994 | £5,670 | -0.226 | Dominated |
| imPulse | £532,507 | 448.956 | £15,192 | -0.264 | Dominated |

ICER=incremental cost effectiveness ratio; QALY=quality adjusted life year

*Algorithm interpretation
