# Supplementary material for: Lead-I ECG for detecting atrial fibrillation in patients attending primary care with an irregular pulse using single-time point testing: A systematic review and economic evaluation
Source: PLoS One. 2019 Dec 23;14(12):e0226671. doi: 10.1371/journal.pone.0226671 (PMC6927656; doi:10.1371/journal.pone.0226671)
Supplement: S1 Text — (DOCX) [file pone.0226671.s018.docx]

## S1 Text. Search strategy (MEDLINE)

1 Lead-I ECG.tw.

2 single lead ECG.tw.

3 (lead I or single lead or automated algorithm).tw.

4 Electrocardiography/

5 (electrocardiog* or ECG).tw.

6 4 or 5

7 3 and 6

8 lead I electrocardiog*.tw.

9 single lead electrocardiog*.tw.

10 1 or 2 or 7 or 8 or 9

11 Atrial Fibrillation/

12 AF.tw.

13 (Atr* adj3 Fibrill*).tw.

14 11 or 12 or 13

15 10 and 14

16 Kardia Mobile.tw.

17 MyDiagnostick.tw.

18 RhythmPad.tw.

19 Zenicor-ECG.tw.

20 imPulse.tw.

21 10 and 20

22 15 or 16 or 17 or 18 or 19 or 21

23 Animals/ not Humans/

24 22 not 23
