# Supplementary material for: Lead-I ECG for detecting atrial fibrillation in patients attending primary care with an irregular pulse using single-time point testing: A systematic review and economic evaluation
Source: PLoS One. 2019 Dec 23;14(12):e0226671. doi: 10.1371/journal.pone.0226671 (PMC6927656; doi:10.1371/journal.pone.0226671)
Supplement: S2 Text — (DOCX) [file pone.0226671.s019.docx]

## S2 Text. Excluded studies

Ineligible intervention (19 studies)

Boyle KO, Morra D, Dorian P, McCrorie A, Haddad P, Taylor L, et al. Atrial fibrillation screening using a handheld ecg device: Results from the heart and stroke foundation (hsf) "be pulse aware" campaign. Stroke 2013;44(12):e184.

Chellappan K, Ab Malek SNH, Jaafar R, Aminuddin A. Self-monitoring technique for stroke prevention among atrial fibrillation patients. International Journal of Stroke 2016;11(Supplement 3):248.

Chen YH, Hung CS, Huang CC, Hung YC, Hwang JJ, Ho YL. Atrial fibrillation screening in nonmetropolitan areas using a telehealth surveillance system with an embedded cloud-computing algorithm: Prospective pilot study. JMIR Mhealth Uhealth 2017;5:e135.

Claes N, Van Laethem C, Goethals M, Goethals P, Mairesse G, Schwagten B, et al. Prevalence of atrial fibrillation in adults participating in a large-scale voluntary screening programme in belgium. Acta Cardiol 2012;67:273-8.

Gilani M, Eklund JM, Makrehchi M. Automated detection of atrial fibrillation episode using novel heart rate variability features. Conf Proc IEEE Eng Med Biol Soc 2016;2016:3461-4.

Hobbs F, Fitzmaurice D, Mant J, Murray E, Jowett S, Bryan S, et al. A randomised controlled trial and cost-effectiveness study of systematic screening (targeted and total population screening) versus routine practice for the detection of atrial fibrillation in people aged 65 and over. The safe study. Health Technol Assess 2005;9(40):iii-iv, ix-x, 1-74.

Kaleschke G, Hoffmann B, Drewitz I, Steinbeck G, Naebauer M, Goette A, et al. Prospective, multicentre validation of a simple, patient-operated electrocardiographic system for the detection of arrhythmias and electrocardiographic changes. Europace 2009;11:1362-8.

Kearley K, Selwood M, Van den Bruel A, Thompson M, Mant D, Hobbs FR, et al. Triage tests for identifying atrial fibrillation in primary care: A diagnostic accuracy study comparing single-lead ecg and modified bp monitors. BMJ Open 2014;4:e004565.

Mant J, Fitzmaurice DA, Hobbs FDR, Jowett S, Murray ET, Holder R, et al. Accuracy of diagnosing atrial fibrillation on electrocardiogram by primary care practitioners and interpretative diagnostic software: Analysis of data from screening for atrial fibrillation in the elderly (safe) trial. BMJ 2007;335:380-2.

McManus DD, Lee J, Maitas O, Esa N, Pidikiti R, Carlucci A, et al. A novel application for the detection of an irregular pulse using an iphone 4s in patients with atrial fibrillation. Heart Rhythm 2013;10:315-9.

McManus D, Chong JW, Soni A, Saczynski JS, Esa N, Napolitano C, et al. Pulse-smart: Pulse-based arrhythmia discrimination using a novel smartphone application. J Cardiovasc Electrophysiol 2016;27:51-7.

Mortelmans C, Van Haelst R, Van Der Auwera J, Grieten L, Vandervoort P, Vaes B. Validation of a new smartphone application for the diagnosis of atrial fibrillation in primary care. Europace 2017;19(Supplement 3):iii16.

Newham WG, Tayebjee MH. Excellent symptom rhythm correlation in patients with palpitations using a novel smartphone based event recorder. J Atr Fibrillation 2017;10:1514.

Proietti M, Mairesse GH, Goethals P, Scavee C, Vijgen J, Blankoff I, et al. A population screening programme for atrial fibrillation: A report from the belgian heart rhythm week screening programme. Europace 2016;18:1779-86.

Rajendram R, Patel S, Kale S, Nangalia V. Ability of clinicians trained in intensive care to interpret rhythm strips. Journal of the Intensive Care Society 2014;1:S70-S1.

Sandhu RK, Deif B, Barake W, Agarwal G, Connolly SJ, Dolovich L, et al. Identification of actionable atrial fibrillation using an integrated cardiovascular screening approach in community pharmacies. Heart Rhythm 2016;1:S415-S6.

Somerville S, Somerville J, Croft P, Lewis M. Atrial fibrillation: A comparison of methods to identify cases in general practice. Br J Gen Pract 2000;50:727-9.

Vyas V, Duran J, Ansaripour A, Niedzielko M, Steel A, Bakhai A. Does a 12-lead ecg more reliably detect atrial fibrilation than a rhythm strip only ecg? Value Health 2014;17(7):A485-A6.

Winkler S, Axmann C, Schannor B, Kim S, Leuthold T, Scherf M, et al. Diagnostic accuracy of a new detection algorithm for atrial fibrillation in cardiac telemonitoring with portable electrocardiogram devices. J Electrocardiol 2011;44:460-4.

Ineligible outcomes (7 studies)

Ara F, Crockford C, John I, Kaba RA. Novel galvanised titanium-based ecg technology can reliably detectarrhythmias. Europace 2015;3:iii53.

Chan PH, Wong CK, Pun L, Wong YF, Wong MM, Chu DW, et al. Diagnostic performance of an automatic blood pressure measurement device, microlife watchbp home a, for atrial fibrillation screening in a real-world primary care setting. BMJ Open 2017;7:e013685.

Chung EH, Guise KD. Qtc intervals can be assessed with the alivecor heart monitor in patients on dofetilide for atrial fibrillation. J Electrocardiol 2015; 48:8-9.

Grieten L, Van Der Auwera J, Vandervoort P, Rivero-Ayerza M, Van Herendael H, De Vusser P, et al. Evaluating smartphone based photoplesythmography as a screening solution for atrial fibrillation: A digital tool to detect afib? J Am Coll Cardiol 2017;69(11 Supplement 1):2499.

Jacobs MS, Kaasenbrood F, Postma MJ, Van Hulst M, Tieleman RG. Cost-effectiveness of screening for atrial fibrillation in primary care with a handheld, single-lead electrocardiogram device in the netherlands. Europace 2018;20:12-8.

Khanbhai ZM, Manning SE, Hussain W. Community pharmacist led atrial fibrillation screening program has the potential to improve atrial fibrillation detection rates and reduce stroke risk. Circulation Conference: American Heart Association's 2016;134.

Mehta DD, Nazir NT, Trohman RG, Volgman AS. Single-lead portable ecg devices: Perceptions and clinical accuracy compared to conventional cardiac monitoring. J Electrocardiol 2015;48:710-6.

Ineligible language (1 study)

Reimert M, Verhoeven A. Screening for atrial fibrillation with single-lead hand-held ecg. Huisarts en Wetenschap 2017;60:474.
