# Supplementary material for: Lead-I ECG for detecting atrial fibrillation in patients attending primary care with an irregular pulse using single-time point testing: A systematic review and economic evaluation
Source: PLoS One. 2019 Dec 23;14(12):e0226671. doi: 10.1371/journal.pone.0226671 (PMC6927656; doi:10.1371/journal.pone.0226671)
Supplement: S3 Text — (DOCX) [file pone.0226671.s020.docx]

## S3 Text. References that appear only in Supplementary Information

56. Sterne JA, Bodalia PN, Bryden PA, Davies PA, Lopez-Lopez JA, Okoli GN, et al. Oral anticoagulants for primary prevention, treatment and secondary prevention of venous thromboembolic disease, and for prevention of stroke in atrial fibrillation: systematic review, network meta-analysis and cost-effectiveness analysis. Health Technol Assess 2017;21(9):1-386

57. Office for National Statistics. Estimates of the population for the UK, England and Wales, Scotland and Northern Ireland - office for national statistics. 2018; Available from: <https://www.ons.gov.uk/peoplepopulationandcommunity/populationandmigration/populationestimates/datasets/populationestimatesforukenglandandwalesscotlandandnorthernireland> [Accessed June 2018].

58. Turakhia MP, Shafrin J, Bognar K, Trocio J, Abdulsattar Y, Wiederkehr D, Goldman DP. Estimated prevalence of undiagnosed atrial fibrillation in the United States. PLoS One 2018;13(4):e0195088.

59. Israel CW, Grönefeld G, Ehrlich JR, Li Y-G, Hohnloser SH. Long-term risk of recurrent atrial fibrillation as documented by an implantable monitoring device: implications for optimal patient care. J Am Coll Cardiol 2004;43:47-52.

60. Kirchhof P, Auricchio A, Bax J, Crijns H, Camm J, Diener H-C, et al. Outcome parameters for trials in atrial fibrillationRecommendations from a consensus conference organized by the German Atrial Fibrillation Competence NETwork and the European Heart Rhythm Association. Europace 2007;9:1006-23.

61. Hobbs F, Fitzmaurice D, Mant J, Murray E, Jowett S, Bryan S, et al. A randomised controlled trial and cost-effectiveness study of systematic screening (targeted and total population screening) versus routine practice for the detection of atrial fibrillation in people aged 65 and over. the SAFE study. Health Technol Assess 2005;9(40):iii-iv, ix-x, 1-74.

62. Curtis L BA. Unit costs of health and social care 2017. 2017; Available from: <https://www.pssru.ac.uk/project-pages/unit-costs/> [Accessed June 2018].

63. Department of Health. NHS reference costs 2016/17. 2017; Available from: <https://improvement.nhs.uk/resources/reference-costs/> [Accessed August 2018].

64. Public Health England. First stroke estimates in England: 2007 to 2016. 2018; Available from: <https://www.gov.uk/government/publications/first-stroke-estimates-in-england-2007-to-2016> [Accessed August 2018].

65. Rothwell PM, Coull AJ, Silver LE, Fairhead JF, Giles MF, Lovelock CE, et al. Population-based study of event-rate, incidence, case fatality, and mortality for all acute vascular events in all arterial territories (oxford vascular study). Lancet Oncol 2005;366:1773-83.

66. Mohan KM, Wolfe CD, Rudd AG, Heuschmann PU, Kolominsky-Rabas PL, Grieve AP. Risk and cumulative risk of stroke recurrence: a systematic review and meta-analysis. Stroke 2011;42:1489-94.
