# Supplementary material for: Lead-I ECG for detecting atrial fibrillation in patients attending primary care with an irregular pulse using single-time point testing: A systematic review and economic evaluation
Source: PLoS One. 2019 Dec 23;14(12):e0226671. doi: 10.1371/journal.pone.0226671 (PMC6927656; doi:10.1371/journal.pone.0226671)
Supplement: S1 Scenarios — (DOCX) [file pone.0226671.s021.docx]

## S1 Scenarios. Scenario analyses

Scenario A: Unit cost associated with the lead-I ECG device

Incremental cost effectiveness results from Scenario A, which investigates the impact of removing the unit cost of the lead-I ECG device from the analysis (using 12-lead ECG in primary care, 2 days to 12-lead ECG) are presented below.

Scenario A: Impact of removing the unit cost of the lead-I ECG device from the analysis, incremental cost effectiveness analysis

| Strategy | Costs | QALYs | Incremental Costs | Incremental QALYs | ICER/ QALY gained |
| --- | --- | --- | --- | --- | --- |
| Standard pathway | £514,187 | 447.963 |  |  |  |
| Kardia Mobile | £515,535 | 449.249 | £1,347 | 1.286 | £1,047 |
| Generic lead-I device | £516,348 | 449.246 | £813 | -0.002 | Dominated |
| RhythmPad GP* | £517,336 | 448.573 | £1,802 | -0.676 | Dominated |
| Zenicor-ECG | £517,854 | 449.199 | £2,319 | -0.050 | Dominated |
| MyDiagnostick | £521,143 | 449.024 | £5,608 | -0.225 | Dominated |
| imPulse | £530,657 | 448.987 | £15,123 | -0.262 | Dominated |

ICER=incremental cost effectiveness ratio; QALY=quality adjusted life year

*Algorithm interpretation

Scenario B: Alternative sensitivity and specificity estimates for MyDiagnostick

Pairwise cost effectiveness results from Scenario B, which investigates the impact of using the sensitivity and specificity estimates based on interpretation of the MyDiagnostick lead-I ECG trace by EP2 (using 12-lead ECG in primary care, 2 days to 12-lead ECG), are presented below.

Scenario B: Impact of using the sensitivity and specificity estimates based on interpretation of the MyDiagnostick lead-I ECG trace by EP2, incremental cost effectiveness analysis

| Strategy | Costs | QALYs | Incremental Costs | Incremental QALYs | ICER/ QALY gained |
| --- | --- | --- | --- | --- | --- |
| MyDiagnostick | £513,623 | 448.898 |  |  |  |
| Standard pathway | £514,187 | 447.963 | £565 | -0.9359 | Dominated |
| Kardia Mobile | £515,551 | 449.249 | £1,928 | 0.3504 | £5,503 |
| Generic lead-I device | £516,730 | 449.246 | £1,179 | -0.0025 | Dominated |
| RhythmPad GP* | £518,436 | 448.573 | £2,885 | -0.6759 | Dominated |
| Zenicor-ECG | £518,468 | 449.199 | £2,917 | -0.0499 | Dominated |
| imPulse | £530,745 | 448.987 | £15,194 | -0.2620 | Dominated |

ICER=incremental cost effectiveness ratio; QALY=quality adjusted life year

*Algorithm interpretation

Scenario C: Diagnosis without 12-Lead ECG/holter monitor

Incremental cost effectiveness results from scenario C which investigates the impact of removing 12-lead ECG and holter monitoring from the lead-I ECG diagnostic pathway (compared to using 12-lead ECG in primary care, 2 days to 12-lead ECG) are presented below.

Scenario C: Impact of removing 12-lead ECG and holter monitoring from the lead-I ECG diagnostic pathway, incremental analysis

| Strategy | Costs | QALYs | Incremental Costs | Incremental QALYs | ICER/ QALY gained |
| --- | --- | --- | --- | --- | --- |
| Standard pathway | £514,187 | 447.963 |  |  |  |
| Kardia Mobile | £515,356 | 448.896 | £1,169 | 0.9335 | £1,252 |
| Generic lead-I device | £516,575 | 448.888 | £1,218 | -0.0085 | Dominated |
| Zenicor-ECG | £519,081 | 448.726 | £3,725 | -0.1697 | Dominated |
| MyDiagnostick | £524,667 | 448.131 | £9,311 | -0.7647 | Dominated |
| RhythmPad GP* | £529,083 | 446.597 | £13,727 | -2.2991 | Dominated |
| imPulse | £534,767 | 448.004 | £19,411 | -0.8924 | Dominated |

ICER=incremental cost effectiveness ratio; QALY=quality adjusted life year

*Algorithm interpretation

Scenario D: 5-year time horizon

Incremental cost effectiveness results from scenario D investigating a 5-year time horizon as a proxy for all undiagnosed patients being identified within 5 years (12-lead ECG in primary care, 2 days to 12-lead ECG) are presented below.

Scenario D: Impact of 5-year time horizon, incremental analysis

| Strategy | Costs | QALYs | Incremental Costs | Incremental QALYs | ICER/ QALY gained |
| --- | --- | --- | --- | --- | --- |
| Standard pathway | £101,668 | 173.979 |  |  |  |
| Kardia Mobile | £102,543 | 174.550 | £876 | 0.5706 | £1,534 |
| Generic lead-I device | £103,234 | 174.549 | £691 | -0.0011 | Dominated |
| Zenicor-ECG | £104,051 | 174.527 | £1,508 | -0.0224 | Dominated |
| RhythmPad GP* | £104,073 | 174.247 | £1,530 | -0.3028 | Dominated |
| MyDiagnostick | £104,774 | 174.449 | £2,231 | -0.1008 | Dominated |
| imPulse | £108,573 | 174.432 | £6,030 | -0.1175 | Dominated |

ICER=incremental cost effectiveness ratio; QALY=quality adjusted life year

*Algorithm interpretation

Scenario E1 to E40: Varying proportion of patients sent for holter testing after lead-I ECG and 12-lead ECG results

Incremental cost effectiveness results from scenarios E1 to E40 exploring the uncertainty in the proportion of people sent for paroxysmal testing following a negative 12-lead ECG result are presented below. Given the complexity of the results, each scenario is only shown for the standard diagnostic pathway compared to Kardia Mobile (the lead-I ECG test was found to be the most cost effective option in the base case analyses) with 12-lead ECG undertaken in primary care and a 2 day wait for a 12-lead ECG.

Scenario E: Varying the percentage of patients sent for holter monitor testing for paroxysmal AF following a negative 12-lead ECG subsequent to the lead-I ECG result, incremental analysis

| Scenario | % of patients being referred for holter monitoring after negative 12-lead ECG | | | Model results | | | | | | |
| --- | --- | --- | --- | --- | --- | --- | --- | --- | --- | --- |
|  | Lead-I pathway | | Standard pathway | Standard pathway | | Lead-I pathway | | Incremental | | ICER per QALY gained |
|  | **Lead-I ECG negative** | **Lead-I ECG positive** |  | **Costs** | **QALYs** | **Costs** | **QALYs** | **Costs** | **QALYs** |  |
| E1 | 0% | 0% | 0% | £515,456 | 447.256 | £513,532 | 449.215 | -£1,924 | 1.959 | Dominates |
| E2 | 0% | 100% | 0% | £515,456 | 447.256 | £513,973 | 449.216 | -£1,482 | 1.959 | Dominates |
| E3 | 0% | 75% | 0% | £515,456 | 447.256 | £513,863 | 449.215 | -£1,593 | 1.959 | Dominates |
| E4 | 0% | 50% | 0% | £515,456 | 447.256 | £513,753 | 449.215 | -£1,703 | 1.959 | Dominates |
| E5 | 0% | 25% | 0% | £515,456 | 447.256 | £513,642 | 449.215 | -£1,813 | 1.959 | Dominates |
| E6 | 25% | 100% | 0% | £515,456 | 447.256 | £514,873 | 449.232 | -£583 | 1.976 | Dominates |
| E7 | 25% | 75% | 0% | £515,456 | 447.256 | £514,762 | 449.232 | -£693 | 1.976 | Dominates |
| E8 | 25% | 50% | 0% | £515,456 | 447.256 | £514,652 | 449.232 | -£804 | 1.976 | Dominates |
| E9 | 25% | 25% | 0% | £515,456 | 447.256 | £514,541 | 449.232 | -£914 | 1.976 | Dominates |
| E10 | 50% | 100% | 0% | £515,456 | 447.256 | £515,772 | 449.249 | £316 | 1.993 | £159 |
| E11 | 50% | 75% | 0% | £515,456 | 447.256 | £515,661 | 449.249 | £206 | 1.993 | £103 |
| E12 | 50% | 50% | 0% | £515,456 | 447.256 | £515,551 | 449.249 | £96 | 1.992 | £48 |
| E13 | 75% | 100% | 0% | £515,456 | 447.256 | £516,671 | 449.266 | £1,215 | 2.010 | £605 |
| E14 | 75% | 75% | 0% | £515,456 | 447.256 | £516,561 | 449.266 | £1,105 | 2.009 | £550 |
| E15 | 100% | 100% | 0% | £515,456 | 447.256 | £517,570 | 449.283 | £2,114 | 2.026 | £1,043 |
| E16 | 0% | 100% | 25% | £514,824 | 447.610 | £513,973 | 449.216 | -£851 | 1.606 | Dominates |
| E17 | 0% | 75% | 25% | £514,824 | 447.610 | £513,863 | 449.215 | -£961 | 1.606 | Dominates |
| E18 | 0% | 50% | 25% | £514,824 | 447.610 | £513,753 | 449.215 | -£1,071 | 1.606 | Dominates |
| E19 | 0% | 25% | 25% | £514,824 | 447.610 | £513,642 | 449.215 | -£1,182 | 1.606 | Dominates |
| E20 | 0% | 100% | 50% | £514,187 | 447.963 | £513,973 | 449.216 | -£214 | 1.253 | Dominates |
| E21 | 0% | 75% | 50% | £514,187 | 447.963 | £513,863 | 449.215 | -£324 | 1.253 | Dominates |
| E22 | 0% | 50% | 50% | £514,187 | 447.963 | £513,753 | 449.215 | -£435 | 1.253 | Dominates |
| E23 | 0% | 100% | 75% | £513,545 | 448.315 | £513,973 | 449.216 | £428 | 0.901 | £476 |
| E24 | 0% | 75% | 75% | £513,545 | 448.315 | £513,863 | 449.215 | £318 | 0.900 | £353 |
| E25 | 0% | 100% | 100% | £512,895 | 448.667 | £513,973 | 449.216 | £1,078 | 0.549 | £1,966 |
| E26 | 25% | 25% | 25% | £514,824 | 447.610 | £514,541 | 449.232 | -£282 | 1.622 | Dominates |
| E27 | 50% | 50% | 50% | £514,187 | 447.963 | £515,551 | 449.249 | £1,364 | 1.286 | £1,060 |
| E28 | 50% | 50% | 25% | £514,824 | 447.610 | £515,551 | 449.249 | £727 | 1.639 | £444 |
| E29 | 75% | 75% | 25% | £514,824 | 447.610 | £516,561 | 449.266 | £1,737 | 1.656 | £1,049 |
| E30 | 75% | 75% | 50% | £514,187 | 447.963 | £516,561 | 449.266 | £2,373 | 1.303 | £1,821 |
| E31 | 75% | 75% | 75% | £513,545 | 448.315 | £516,561 | 449.266 | £3,016 | 0.951 | £3,172 |
| E32 | 100% | 100% | 25% | £514,824 | 447.610 | £517,570 | 449.283 | £2,746 | 1.673 | £1,641 |
| E33 | 100% | 100% | 50% | £514,187 | 447.963 | £517,570 | 449.283 | £3,383 | 1.320 | £2,562 |
| E34 | 100% | 100% | 75% | £513,545 | 448.315 | £517,570 | 449.283 | £4,025 | 0.968 | £4,159 |
| E35 | 25% | 50% | 50% | £514,187 | 447.963 | £514,652 | 449.232 | £464 | 1.270 | £366 |
| E36 | 50% | 50% | 75% | £513,545 | 448.315 | £515,551 | 449.249 | £2,006 | 0.934 | £2,148 |
| E37 | 25% | 75% | 75% | £513,545 | 448.315 | £514,762 | 449.232 | £1,217 | 0.917 | £1,327 |
| E38 | 25% | 75% | 75% | £513,545 | 448.315 | £514,762 | 449.232 | £1,217 | 0.917 | £1,327 |
| E39 | 50% | 75% | 75% | £513,545 | 448.315 | £515,661 | 449.249 | £2,116 | 0.934 | £2,266 |
| E40 | 100% | 100% | 100% | £512,895 | 448.667 | £517,570 | 449.283 | £4,675 | 0.616 | £7,594 |

ICER=incremental cost effectiveness ratio; MPP=manual pulse palpation; QALY=quality adjusted life year

Scenario F: Cost of a smartphone or tablet added to the cost of the Kardia Mobile device

In order to perform a lead-I ECG with the Kardia Mobile device, it is necessary to connect the device to a smartphone or tablet. The EAG assumed in the base case that a GP would already have access to a smartphone or tablet that could be used alongside the Kardia Mobile device and would incur no extra cost. The cost of a supplementary smartphone or tablet for use alongside the Kardia Mobile device was investigated in a scenario analysis.

The cost of purchasing a smartphone or tablet varies substantially depending on the type of device, so any estimate of the cost of such a device may not reflect reality for some or any GP practices. The EAG considered it would be justified to perform a threshold analysis to estimate the level at which the extra cost of a supplementary smartphone or tablet would result in Kardia Mobile no longer dominating the other lead-I ECG devices or generating an ICER of £20,000 per QALY gained compared to the standard pathway. The estimated minimum cost of a supplementary smartphone or tablet for Kardia Mobile to no longer dominate ranged from £2,885 versus RhythmPad to £15,194 versus the ImPulse device. Provided a supplementary smartphone or tablet costs less than £24,362, then the ICER per QALY gained for Kardia Mobile compared to the standard pathway would be below £20,000.

The results of the threshold analysis from Scenario F, which calculates the minimum cost of a supplementary smartphone or tablet device that would result in Kardia Mobile no longer being dominant over each of the alternative strategies (using 12-lead ECG in primary care, 2 days to 12-lead ECG) are presented below.

Scenario F: Minimum cost per supplementary smartphone or tablet device for a non-dominant ICER per QALY gained versus Kardia Mobile (the cost to make the ICER £20,000 per QALY for Kardia Mobile versus the standard pathway)

| **Strategy** | **Minimum cost per supplementary device** |
| --- | --- |
|  | ***Kardia Mobile ICER per QALY gained = £20,000*** |
| Standard pathway | £24,362 |
|  | ***Kardia Mobile non-dominant ICER per QALY gained*** |
| RhythmPad GP* | £2,885 |
| Zenicor-ECG | £2,917 |
| MyDiagnostick | £5,682 |
| imPulse | £15,194 |

*Algorithm interpretation

Scenario G: Extending the lifespan of the RhythmPad GP device from 1 year to 3 years

The manufacturer of the RhythmPad GP device gave the minimum projected life of the device as 1 year, with the potential for it to last up to 3 years. Changing the lifespan of the RhythmPad GP from 1 year to 3 years reduces total costs; however, RhythmPad GP remains dominated by the Kardia Mobile device.

Incremental cost effectiveness results from Scenario G, which investigates the impact of extending the lifespan of the Rhythmpad GP device from 1 year to 3 years (using 12-lead ECG in primary care, 2 days to 12-lead ECG) are presented below.

Scenario G: Impact of extending the lifespan of the Rhythmpad GP device from 1 year to 3 years, incremental cost effectiveness analysis

| Strategy | Costs | QALYs | Incremental Costs | Incremental QALYs | ICER/ QALY gained |
| --- | --- | --- | --- | --- | --- |
| Standard pathway | £514,187 | 447.963 |  |  |  |
| Kardia Mobile | £515,551 | 449.249 | £1,364 | 1.2863 | £1,060 |
| RhythmPad GP* | £517,703 | 448.573 | £2,152 | -0.6759 | Dominated |
| Zenicor-ECG | £518,468 | 449.199 | £2,917 | -0.0499 | Dominated |
| MyDiagnostick | £521,233 | 449.024 | £5,682 | -0.2249 | Dominated |
| imPulse | £530,745 | 448.987 | £15,194 | -0.262 | Dominated |

ICER=incremental cost effectiveness ratio; QALY=quality adjusted life year

*Algorithm interpretation

Scenario H: Including a QALY decrement for bleeds

In the base case analysis no disutility for bleeds was assumed as robust estimates on utlity of bleeds could not be identified in the literature. As these are rare events of short duration the impact on QALYs was expected to be minor. To test the impact of the assumption of no QALY loss for bleeds, a value for utility loss and duration of bleed was taken from the apixaban technology appraisal where the company used a disutility for major bleeds of 0.1070 from a standard gamble exercise of patients with AF valuing different health outcomes and AEs that could hypothetically occur whilst taking anticoagulation. The company in the apixaban appraisal assumed that major bleeds would last for 14 days; this was a company assumption and no justification was provided. Applying the duration of bleed to the utility loss and assuming all bleeds are major means each bleed results in a 0.004 QALY loss. The impact of introducing a disutility for bleeds in the model (using 12-lead ECG in primary care, 2 days to 12-lead ECG) are presented below. As can be seen, whilst the standard pathway and lead-I devices all lose QALYs as expected, as the total lifetime number of bleeds for the cohort of patients in the model was only 0.017 higher with Kardia Mobile compared to the standard pathway and the QALY loss from bleeds was so small, the impact on incremental QALYs was almost zero and so the introduction of a disutility for bleeds did not affect the ICER per QALY gained.

Scenario H: Impact of assuming a QALY loss from bleeds, incremental cost effectiveness analysis

| Strategy | Costs | QALYs | Incremental Costs | Incremental QALYs | ICER/ QALY gained |
| --- | --- | --- | --- | --- | --- |
| Standard pathway | £514,187 | 447.901 |  |  |  |
| Kardia Mobile | £515,551 | 449.187 | £1,364 | 1.286 | £1,060 |
| RhythmPad GP* | £518,436 | 448.511 | £2,885 | -0.676 | Dominated |
| Zenicor-ECG | £518,468 | 449.137 | £2,917 | -0.050 | Dominated |
| MyDiagnostick | £521,233 | 448.962 | £5,682 | -0.225 | Dominated |
| imPulse | £530,745 | 448.925 | £15,194 | -0.262 | Dominated |

ICER=incremental cost effectiveness ratio; QALY=quality adjusted life year

*Algorithm interpretation

Scenario I: Using alternative sensitivity and specificity estimates for Kardia Mobile from the pooled analysis with interpretation of the trace by EP2

Incremental deterministic and probablisitic cost effectiveness results from Scenario I, which investigates the impact of using the sensitivity and specificity estimates based on interpretation of the Kardia Mobile lead-I ECG trace by EP2 (using 12-lead ECG in primary care, 2 days to 12-lead ECG) are presented below. The CEAC for Scenario I is presented in the Figure below.

Scenario I: Impact of using the sensitivity and specificity estimates based on interpretation of the Kardia Mobile lead-I ECG trace by EP2, incremental deterministic cost effectiveness analysis

| Strategy | Costs | QALYs | Incremental Costs | Incremental QALYs | ICER/ QALY gained |
| --- | --- | --- | --- | --- | --- |
| Kardia Mobile | £514,177 | 449.181 |  |  |  |
| Standard pathway | £514,187 | 447.963 | £10 | -1.219 | Dominated |
| RhythmPad GP* | £518,436 | 448.573 | £4,259 | -0.608 | Dominated |
| Zenicor-ECG | £518,468 | 449.199 | £4,290 | 0.018 | £242,994 |
| MyDiagnostick | £521,233 | 449.024 | £2,765 | -0.175 | Dominated |
| imPulse | £530,745 | 448.987 | £12,277 | -0.212 | Dominated |

ICER=incremental cost effectiveness ratio; QALY=quality adjusted life year

*Algorithm interpretation

Scenario I: Impact of using the sensitivity and specificity estimates based on interpretation of the Kardia Mobile lead-I ECG trace by EP2, incremental probabilistic cost effectiveness analysis

| Strategy | Costs | QALYs | Incremental Costs | Incremental QALYs | ICER/ QALY gained |
| --- | --- | --- | --- | --- | --- |
| Kardia Mobile | £521,903 | 455.16065 |  |  |  |
| Standard pathway | £522,204 | 453.96612 | £301 | -1.1945 | Dominated |
| RhythmPad GP* | £526,453 | 454.56963 | £1,798 | -0.5910 | Dominated |
| Zenicor | £526,518 | 455.17774 | £1,864 | 0.0171 | £109,012 |
| MyDiagnostick | £529,316 | 455.00675 | £4,661 | -0.1710 | Dominated |
| ImPulse | £538,857 | 454.97117 | £14,203 | -0.2066 | Dominated |

ICER=incremental cost effectiveness ratio; QALY=quality adjusted life year

*Algorithm interpretation


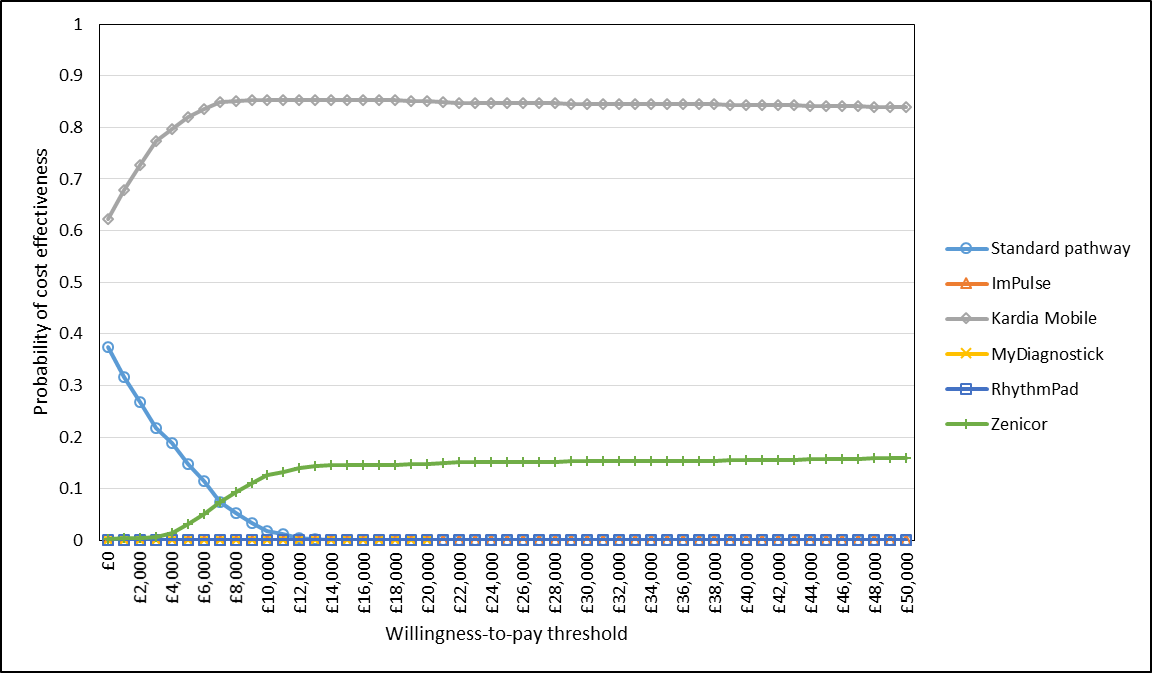


CEAC Scenario I: all lead-I devices

Scenario J: Assuming that the rates of HS for people treated with NOACs who do not have AF are the same as the rates of HS for people treated with NOACs who have AF

Incremental cost effectiveness results from Scenario J, which investigates the impact of assuming that rates of HS for people treated with NOACs who do not have AF are the same as the rates of HS for people treated with NOACs who have AF (using 12-lead ECG in primary care, 2 days to 12-lead ECG) are presented below.

Scenario J: Impact of assuming that rates of HS are the same for people treated with NOACs who do not have AF as the rates of HS for people treated with NOACs who have AF, incremental cost effectiveness analysis

| Strategy | Costs | QALYs | Incremental Costs | Incremental QALYs | ICER/ QALY gained |
| --- | --- | --- | --- | --- | --- |
| Standard pathway | £514,187 | 447.963 |  |  |  |
| Kardia Mobile | £516,109 | 448.697 | £1,922 | 0.734 | £2,618 |
| RhythmPad GP* | £518,957 | 448.055 | £2,848 | -0.642 | Dominated |
| Zenicor-ECG | £519,177 | 448.511 | £3,068 | -0.186 | Dominated |
| MyDiagnostick | £522,133 | 448.166 | £6,023 | -0.530 | Dominated |
| imPulse | £532,320 | 447.537 | £16,211 | -1.159 | Dominated |

ICER=incremental cost effectiveness ratio; QALY=quality adjusted life year

*Algorithm interpretation
